# Low-grade peripheral inflammation affects brain pathology in the *App*^*NL-G-F*^mouse model of Alzheimer’s disease

**DOI:** 10.1186/s40478-021-01253-z

**Published:** 2021-10-07

**Authors:** Junhua Xie, Nina Gorlé, Charysse Vandendriessche, Griet Van Imschoot, Elien Van Wonterghem, Caroline Van Cauwenberghe, Eef Parthoens, Evelien Van Hamme, Saskia Lippens, Lien Van Hoecke, Roosmarijn E. Vandenbroucke

**Affiliations:** 1 VIB-UGent Center for Inflammation Research, Technologiepark-Zwijnaarde 71, 9052 Ghent, Belgium; 2 Department of Biomedical Molecular Biology, Ghent University, 9000 Ghent, Belgium; 3 VIB BioImaging Core, VIB, Ghent, Belgium

**Keywords:** Low-grade peripheral inflammation, Brain barriers, Choroid plexus, Blood-CSF barrier, Alzheimer's disease

## Abstract

Alzheimer’s disease (AD) is a chronic neurodegenerative disease characterized by the accumulation of amyloid β (Aβ) and neurofibrillary tangles. The last decade, it became increasingly clear that neuroinflammation plays a key role in both the initiation and progression of AD. Moreover, also the presence of peripheral inflammation has been extensively documented. However, it is still ambiguous whether this observed inflammation is cause or consequence of AD pathogenesis. Recently, this has been studied using amyloid precursor protein (APP) overexpression mouse models of AD. However, the findings might be confounded by APP-overexpression artifacts. Here, we investigated the effect of low-grade peripheral inflammation in the APP knock-in (*App*^*NL-G-F*^) mouse model. This revealed that low-grade peripheral inflammation affects (1) microglia characteristics, (2) blood-cerebrospinal fluid barrier integrity, (3) peripheral immune cell infiltration and (4) Aβ deposition in the brain. Next, we identified mechanisms that might cause this effect on AD pathology, more precisely Aβ efflux, persistent microglial activation and insufficient Aβ clearance, neuronal dysfunction and promotion of Aβ aggregation. Our results further strengthen the believe that even low-grade peripheral inflammation has detrimental effects on AD progression and may further reinforce the idea to modulate peripheral inflammation as a therapeutic strategy for AD.

**Supplementary Information:**

The online version contains supplementary material available at 10.1186/s40478-021-01253-z.

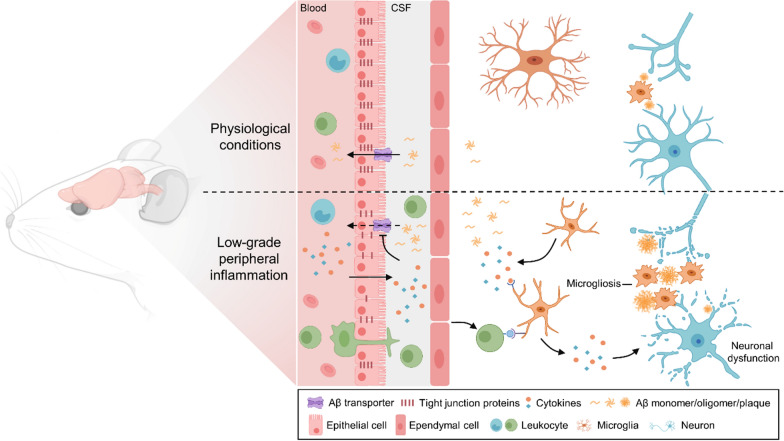

## Introduction

Alzheimer’s disease (AD) is a devastating age-related neurodegenerative disorder that is characterized by the progressive and disabling deficits in cognitive functions including reasoning, attention, judgment, comprehension, memory and language. AD is the most common form of dementia and may contribute to 60–70% of cases [1]. Worldwide, nearly 50 million people have AD or related dementia, and this number will multiply in the next decades [1]. The speed of disease progression is subjective to individual variability, but patients are estimated to live from a few up to 20 years after their diagnosis [1]. Next to dramatically affecting the life quality and expectancy of patients, the disease also takes its toll on our healthcare system and is becoming one of the most economically taxing diseases in developed countries. Unfortunately, only symptomatic medication that is effective for some AD patients is available, but no cure nor treatment to reverse or even halt disease progression exists.

It is already well-known for decades that the deposition of amyloid-beta (Aβ) protein in senile plaques outside neurons and the formation of neurofibrillary tangles (NFT) composed of hyperphosphorylated Tau (p-Tau) protein inside neurons result in the loss of synapses and neurodegeneration which ultimately leads to symptoms associated with AD [12]. The steady progress in the understanding of the etiopathogenesis has led to the evaluation of therapies aiming to reduce pathological aggregates of either Aβ or p-Tau. Unfortunately, none of these strategies has led to clinical success [10]. As a consequence, a more in-depth and more comprehensive understanding of the AD pathology is crucial for the development of novel effective therapies.

Recently, emerging evidence suggests that innate immune activation plays a crucial role in the pathogenesis and progression of AD [14, 24, 35]. For example, genome-wide association studies (GWAS) have demonstrated that genes for immune receptors including *CR1*, *CLU*, *CD33*, and *TREM2* are associated with AD development[26]. More recently, Sierksma et al. also identified *SYK*, *GRN*, *SLC2A5*, *PYDC1*, *HEXB*, and *BLNK* as risk genes[59]. All these identified risk genes are involved in the regulation of the immune response within the central nervous system (CNS) but remarkably also outside the CNS. Moreover, epidemiological and translational research suggests that peripheral inflammation may promote AD pathology [67]. All these findings support a substantial involvement of both peripheral and central immune function in AD pathogenesis. Consequently, understanding the connections between the immune system and AD development might be key in our search for therapies against AD.

The principal resident immune cell of the CNS is the microglia. These phagocytotic cells are ubiquitously distributed in the brain and patrol their assigned brain regions for the presence of pathogens and cell debris [23]. Moreover, microglial cells provide factors that support overall tissue maintenance and plasticity of neuronal circuits [32]. However, when homeostasis is disrupted, e.g. in response to inflammation, microglia adopt an activated state which is characterized by an amoeba-like structure, an increase in proinflammatory cytokine expression and a higher phagocytic activity [64]. If such an imbalance in homeostasis persists, the microglia cells trigger an exaggerated inflammatory response leading to a sustained exposure of neurons to pro-inflammatory mediators, with neuronal dysfunction and cell death as a consequence [25]. During aging and neurodegeneration, microglia show enhanced sensitivity to inflammatory stimuli, so called priming of the microglial cells. Microglia might also be primed in response to peripheral immune reaction [64].

While numerous studies have looked into the presence of inflammation in AD using mouse models and patient samples [46], only a limited amount of studies have looked into the direct impact of peripheral inflammation on AD pathology [29, 31, 68]. Among them is the recent publication of Tejera et al*.* showing that peripheral inflammation alters Aβ pathology by negatively regulating microglial clearance capacity [64]. Importantly, all research performed so far on the effect of peripheral inflammation on AD pathology is performed in mouse models that overexpress the Aβ precursor protein (APP) in combinations with different familial AD (FAD) associated mutations in APP or presenilin 1 (PS1), such as Tg2576, APP/PS1, 5xFAD and 3xTg-AD [53]. These so called first-generation transgenic mouse models exhibit AD pathology, but the overexpression may cause additional phenotypes unrelated to AD. In addition, the mouse models use the neuron-specific Thy1-promotor to overexpress the N-terminal truncated Aβ species, which makes APP processing even less similar to the human situation. Contrary, second-generation mouse models utilize an APP knock-in strategy that closer represents the physiological accumulation of Aβ without phenotypes related to overexpression [52]. In this study, we are the first to report on the effects of low-grade peripheral inflammation in the more representative second-generation mouse models for AD, namely the *App*^*NL-G-F*^ mouse model. Our data reveal that, in agreement with the study of Tejera et al*.* [64], microglial activity is also affected using this AD mouse model. Besides, our results reveal that upon low-grade peripheral inflammation there is an influx of myeloid cells into the brain and a disruption of the blood-cerebrospinal fluid (CSF) barrier. Moreover, we demonstrate that not only microglial Aβ clearance is affected by low-grade peripheral inflammation but also Aβ transport across the brain barriers and neuronal functioning.

## Results

### Low-grade peripheral inflammation induces neuroinflammation in second-generation Alzheimer’s disease mouse model.

**Fig. 1:**
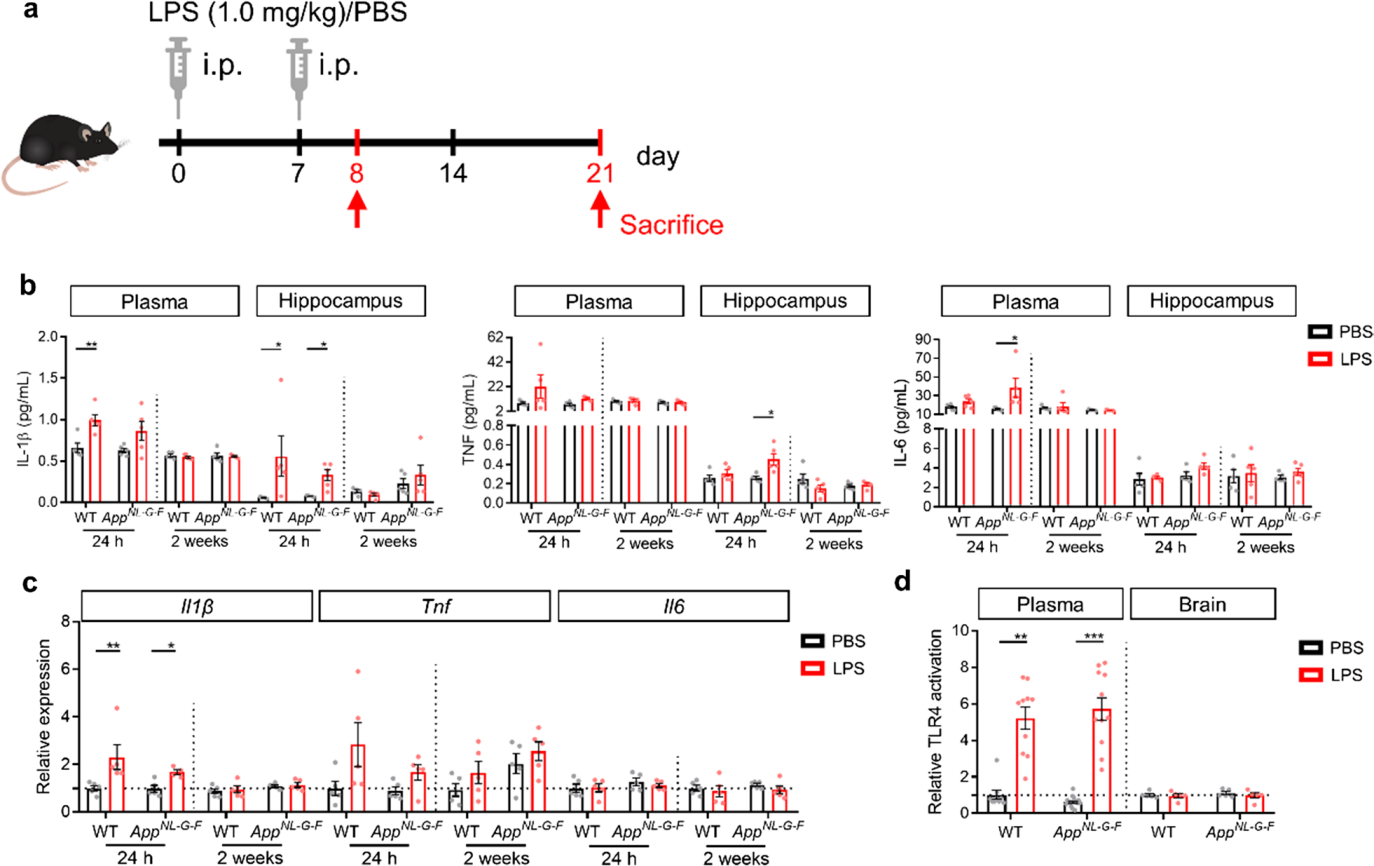
LPS induces transient peripheral inflammation and neuroinflammation. **a** Schematic representation of the experimental design. **b** Protein levels of IL-1β, TNF and IL-6 in plasma and hippocampus (n = 5–10). **c** Expression of the pro-inflammatory genes *Il1β*, *Tnf* and *Il6* in hippocampus (n = 5–10). **d** Analysis of relative TLR4 activation by plasma and brain lysate (n = 9–21). Mean ± SEM, two-way ANOVA Bonferroni’s post hoc test for multiple comparisons. *p < 0.05, **p < 0.01, ***p < 0.001

During the last years it became increasingly clear that the induction of peripheral inflammation causes an immune response in the CNS [48, 54, 55], while its impact on *e.g.* Alzheimer-like pathology is less well studied. Here, we used two i.p. injections (day 0 and day 7) of a low LPS dose (1.0 mg/kg body weight) to study how low-grade peripheral inflammation may affect AD pathology in 20–23 weeks old *App*^*NL-G-F*^ mice 24 h (day 8) and 2 weeks (day 21) after the last LPS injection (Fig. 1a).

Both WT and* App*^*NL-G-F*^ mice show a drop in body weight and an increase in plasma IL-6 levels in response to both LPS injections (Additional file 4: Fig. 1). The IL-6 response is slightly more pronounced in the* App*^*NL-G-F*^ compared to the wild type mice, and in all case we observed a less strong inflammatory response to the second LPS injection. Figure 1b shows elevated levels of the pro-inflammatory cytokines IL-1β, TNF and IL-6 in the plasma 24 h after LPS injection (Fig. 1b). In contrast, all cytokines were back at baseline levels 2 weeks later. Also, neuroinflammation was observed by an increase in protein and mRNA levels of IL-1β and TNF, but not IL-6, in the hippocampus (Fig. 1b, c). Similar to the peripheral cytokine levels, also the increase in brain cytokine levels was transient as this increase was detectable 24 h after LPS injection while the levels were normalized both in WT mice and *App*^*NL-G-F*^ mice 2 weeks later. The increase in brain inflammation at 24 h is not due to the presence of LPS in the brain as we could not detect TLR4 activation in brain lysates, despite the fact that LPS is still present in the blood at that timepoint (Fig. 1d).

Next, we looked into the effect of low-grade peripheral inflammation on neuropathological changes in both WT and *App*^*NL-G-F*^ mice. We investigated the effect on (1) microglia characteristics, (2) influx in the CNS of peripheral myeloid cells and (3) integrity of the blood-CSF barrier.

### Low-grade peripheral inflammation affects microglia characteristics.

**Fig. 2:**
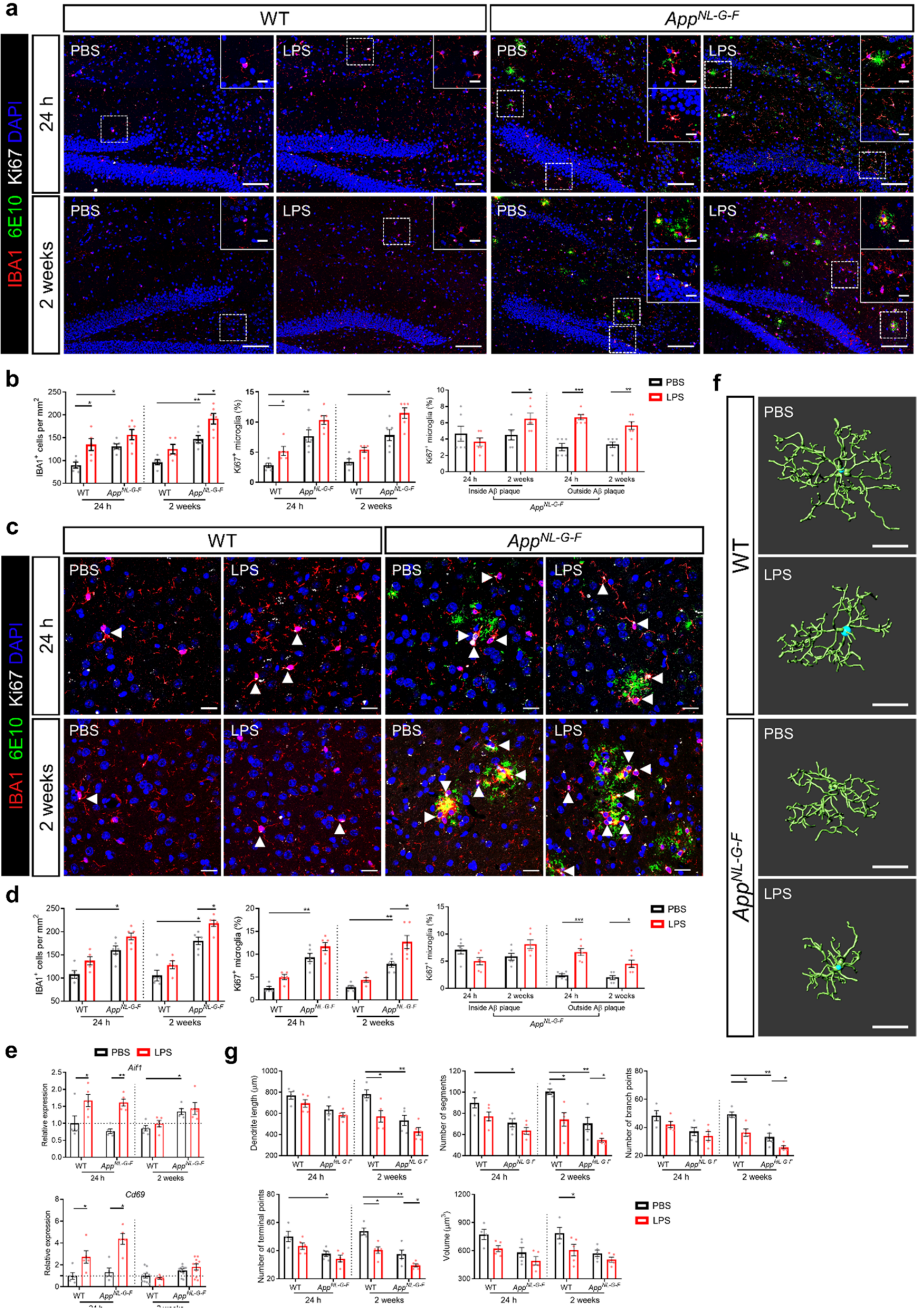
Low-grade peripheral inflammation affects microglia proliferation and activation. **a** Representative images of IBA1, 6E10 and Ki67 staining in hippocampus. Scale bar: 100 μm and 20 μm (insert). **b** Quantification of microglial proliferation in hippocampus (n = 4–6). **c** Representative images of IBA1, 6E10 and Ki67 staining in cortex. Scale bar: 20 μm. **d** Quantification of microglial proliferation in cortex (n = 4–6). **d** Gene expression of microgliosis marker *Aif1* and *Cd69* in hippocampus (n = 5–10). **f** Representative 3D reconstruction images of IBA1^+^ microglia from hippocampus 2 weeks after LPS stimulation. Scale bars: 20 µm. **g** Imaris-based quantification of cell morphology of IBA1^+^ microglia in hippocampus. Each symbol represents one mouse, each mouse was randomly selected with 3–5 cells outside the Aβ for analysis (n = 4–5). Mean ± SEM, two-way ANOVA Bonferroni’s post hoc test for multiple comparisons. *p < 0.05, **p < 0.01

Microglia, the brain-resident immune cells, are the key players in regulating central inflammation. Tejera et al. recently described that microglial cells of WT mice show morphological signs of activation 24 h after LPS injection [64]. Here, we elaborated further on the effect of low-grade peripheral inflammation on brain inflammation in WT mice and *App*^*NL-G-F*^ mice by studying microglia proliferation and activation. Moreover, we compared microglia characteristics when peripheral inflammation is still ongoing (24 h after the last low dose LPS injection) to when peripheral inflammation is resolved (2 weeks after the last LPS injection). Our analysis revealed more microglia and increased proliferation in * App *^*NL-G-F*^ mice compared to WT mice at baseline. Moreover, LPS challenge further increases both of these parameters. In addition, the relative effect of LPS on WT and* App*^*NL-G-F*^ mice is similar, but LPS stimulated* App*^*NL-G-F*^ mice display with the highest number of IBA1 + microglia cells, the highest rate of proliferation, the shortest dendrite length, the smallest number of branch points and the smallest volume. All of this is consistent with the increasing number of microglia and their amoeboid stage (Fig. 2a–d). The significant increase of Ki67^+^ microglial cells was only observed in the cortex of *App*^*NL-G-F*^ mice 2 weeks after peripheral inflammation and in the hippocampus of WT mice 24 h after the last LPS injection (Fig. 2a–d). Especially microglial cells that are not located in the area of Aβ plaques showed a high proliferation upon peripheral inflammation (Fig. 2a–d).

Also, the microgliosis marker Aif1 and the microglial activation marker CD69 were significantly upregulated in the hippocampus 24 h after LPS treatment both in WT and in *App*^*NL-G-F*^ mice (Fig. 2e). This increase is transient as no differences were observed 2 weeks after the last LPS treatment.

Despite the fact that peripheral inflammation and neuroinflammation are not detectable 2 weeks after LPS injection based on cytokine levels (Fig. 1b), the quantitative morphometric three-dimensional (3D) measurements of IBA1^+^ microglial cells revealed a significant decrease in the number of segments, branch and terminal points in hippocampus of WT and *App*^*NL-G-F*^ mice 2 weeks after peripheral inflammation compared to their respective controls (Fig. 2f, g). However, the shorter processes and smaller volumes were only observed in WT mice injected with LPS compared to WT mice injected with PBS. Interestingly, the *App*^*NL-G-F*^ mice also showed more activated microglia compared with WT mice in basal conditions (Fig. 2g). Additionally, we also investigated the morphological changes of microglia in the cortex (Additional file 4: Fig. 2). WT mice showed more pronounced changes in all the examined parameters, namely dendrite length, number of segments, branch and terminal points and cell volume 2 weeks after the last LPS injections compared to the control condition, while this was not observed in *App*^*NL-G-F*^ mice.

### Low-grade peripheral inflammation induces leukocyte trafficking to the brain

**Fig. 3:**
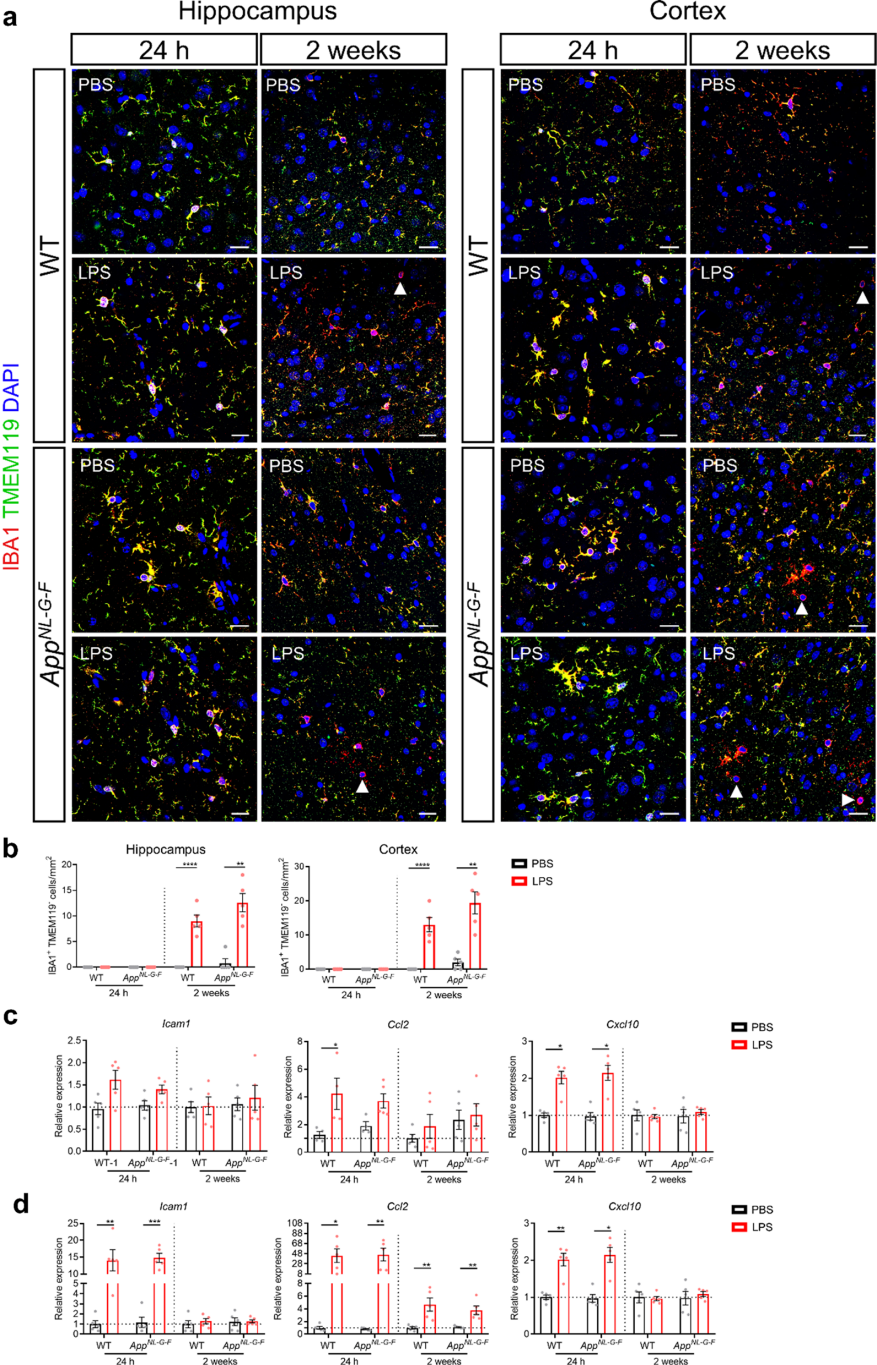
Low-grade peripheral inflammation induces leukocytes trafficking to the brain. **a** Representative images of IBA1 and TMEM119 staining in hippocampus and cortex. Scale bar: 20 μm. **b** Quantification of IBA1^+^ TMEM119^-^ macrophages in hippocampus (left graph) and cortex (right graph). Each symbol represents one mouse. Expression of the gene *Icam1, Ccl2* and *Cxcl10* in hippocampus **c** and in CP **d**. Mean of 5 ± SEM, two-way ANOVA Bonferroni’s post hoc test for multiple comparisons. *p < 0.05, **p < 0.01, ****p < 0.0001

Next to brain resident microglial cells, also infiltrating leukocytes can contribute to neuroinflammation. To investigate the infiltration of peripheral macrophages in the brain after LPS injection, brain sections were immunostained for IBA1 and TMEM119. TMEM119 is specifically expressed on microglial cells, but not on IBA1^+^ infiltrating macrophages [16, 22]. As shown in Fig. 3a, b, no macrophage infiltration was observed in the cortex or hippocampus 24 h after LPS stimulation, neither in WT nor *App*^*NL-G-F*^ mice. In contrast, 2 weeks later, a clear increase in infiltrating macrophages was visible in LPS stimulated mice compared to control mice. This was again observed in both WT and* App*^*NL-G-F*^ mice. Although we observed an increased infiltration of peripheral immune cells into the brain upon LPS challenges, the infiltration is still very modest. In addition, perivascular macrophages (PVMs) are also IBA1^+^ and TMEM119^−^ and these cells have been shown to increase in neurodegenerative disease models [20]. Therefore, other techniques which can accurately distinguish invading peripheral monocytes from brain endogenous PVMs and microglia, for example using genetic labeling of different myeloid populations [49], should be used to further validate this result.

Furthermore, we checked the expression of leukocyte trafficking molecules in the brain during peripheral inflammation and we show that exposure to LPS significantly increases the expression levels of integrin ligand (*Icam1)* and chemokines (*Ccl2* and *Cxcl10)* in the hippocampus and/or choroid plexus (CP) of WT and *App*^*NL-G-F*^ mice 24 h after the second LPS injection. However, only *Ccl2* expression in the CP remained increased 2 weeks after LPS stimulation. In addition, the gene expression of *Icam1*, *Ccl2* and *Cxcl10* was more significantly upregulated in response to low-grade peripheral inflammation in the CP compared to the hippocampus (Fig. 3c, d). Taken together, these findings indicate that low-grade peripheral inflammation induces immune cell infiltration into the brain.

### Loss of blood-CSF barrier integrity in response to low-grade peripheral inflammation

**Fig. 4:**
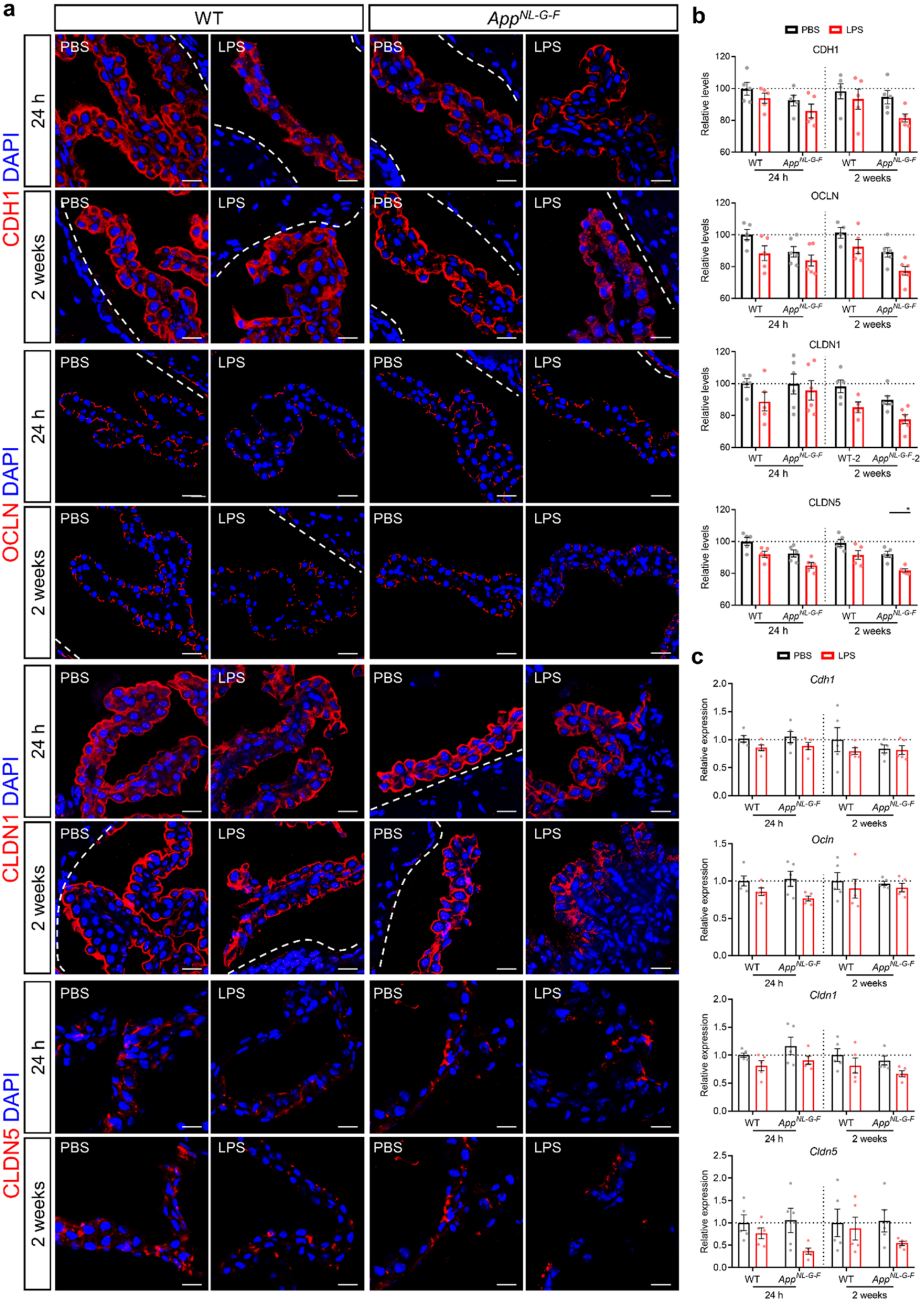
Characterization blood-CSF barrier integrity during peripheral immune challenge. **a**, **b** Representative images of CP and quantification of the percentage red staining of stained for CDH1, OCLN, CLDN1 and CLDN5. The dotted line indicates the ependymal cells that line the ventricle (n = 4–6). Scale bar: 20 μm. **c** Expression of the genes *Cdh1*, *Ocln*, *Cldn1* and *Cldn5* in hippocampus (n = 5). Mean ± SEM, two-way ANOVA Bonferroni’s post hoc test for multiple comparisons. *p < 0.05

We have previously shown that high dose LPS has detrimental effects on blood-CSF barrier integrity, while the effect on blood–brain barrier (BBB) was less pronounced [2, 66]. To investigate whether low-grade peripheral inflammation alters the tight junction (TJ) complex at the CP epithelial cells, the cellular localization of the TJ proteins was evaluated by immunocytochemistry and confocal microscopy. In control conditions, E-cadherin (CDH1), Occludin (OCLN), Claudin-1 (CLDN1) and Claudin-5 (CLDN5) immunoreactivity appeared as a near continuous staining at the apical cell border (Fig. 4a). Upon low-grade peripheral inflammation, a loss of these TJ proteins immunostaining was observed, leading to a fragmented border and diffuse distribution staining, although the effect on *App*^*NL-G-F*^ mice was more pronounced (Fig. 4a). In addition, immunofluorescence quantitative analysis confirmed lower expression of these TJ proteins in the CP of LPS injected WT and *App*^*NL-G-F*^ mice compared with the corresponding PBS injected mice. However, the lower expression of TJ proteins only showed a significant downregulation of CLDN5 2 weeks after LPS injection in *App*^*NL-G-F*^ mice (Fig. 4b). Consistently, the gene expression levels showed downregulation trends after LPS challenge and the *App*^*NL-G-F*^ mice seemed more susceptible to disruption by systemic LPS than the WT mice (Fig. 4c). These results show that peripheral inflammation has more pronounced effects on redistribution of TJ proteins and less on their gene expression. Additionally, we also investigated the TJ protein ZO-1 but no differences were visible either on protein level nor on mRNA level, at least not at the examined time points (Additional file 4: Fig. 3a-c). Collectively, these results suggest that the blood-CSF barrier in *App*^*NL-G-F*^ mice is more vulnerable to low-grade peripheral inflammation compared to their WT counterparts.

IL-1β is a major pro-inflammatory cytokine released from activated microglia and have been demonstrated that IL-1β treatment increases BBB permeability in vitro [69]. Here, we hypothesize that IL-1β may also directly affect blood-CSF barrier permeability. To this end, we studied the effect of IL-1β on blood-CSF barrier integrity using primary CP epithelial cells followed by transepithelial electrical resistance (TEER) measurements and TJ proteins staining. This revealed that IL-1β treatment significantly reduced TEER and TJ proteins expression including ZO-1 and OCLN (Additional file 4: Fig. 4a-c). Also, a fragmented border staining of ZO-1 and OCLN was observed upon IL-1β treatment (Additional file 4: Fig. 4b). These results suggest that the proinflammatory cytokine IL-1β is sufficient to induce loss of blood-CSF barrier integrity, but only induces limited differences in the expression of TJ proteins and genes in primary CP epithelial cells.

### Low-grade peripheral inflammation results in a higher amyloid deposition over time in *App*^***NL-G-F***^*** mice***

It is already well-known for decades that the deposition of Aβ protein in senile plaques outside neurons results in the loss of synapses and neurodegeneration which ultimately leads to symptoms associated with AD [12]. Next to this, peripheral inflammation has been associated with AD [28]. Here, we analyzed whether low-grade inflammation has an impact on Aβ pathology in *App*^*NL-G-F*^ mice.

**Fig. 5:**
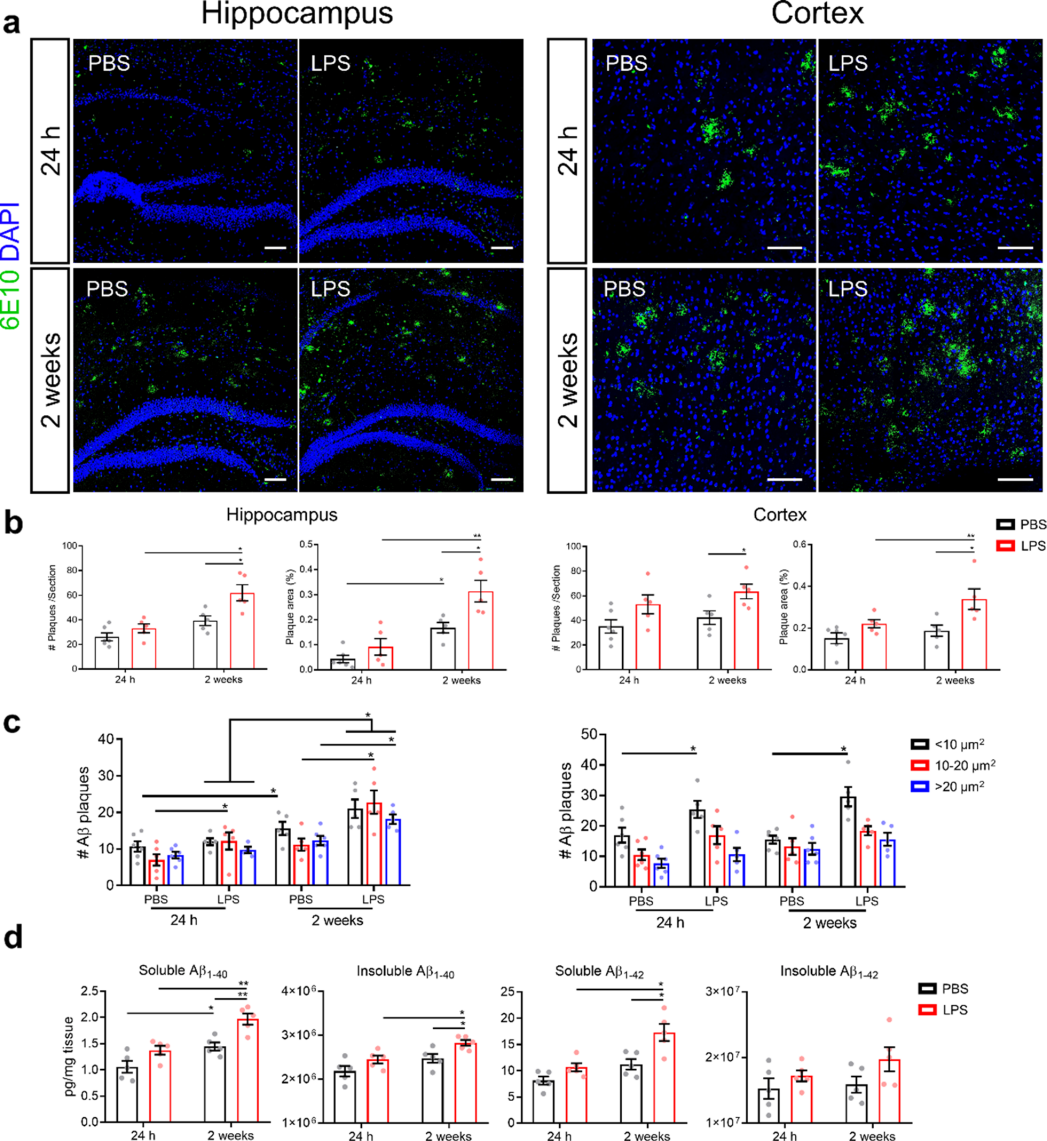
Low-grade peripheral inflammation affects Aβ deposition in *App*^*NL-G-F*^ mice. **a** Representative images of 6E10 staining in hippocampus and cortex of *App*^*NL-G-F*^ mice. Scale bar: 100 μm. **b** Quantification of Aβ plaque area and number (n = 5–6). Hippocampus (left two graphs); cortex (right two graphs). **c** Quantification of Aβ plaque size distribution. Hippocampus (left); cortex (right); (n = 5–6). **d** Soluble and insoluble Aβ_1-40_ and Aβ_1-42_ levels in prefrontal cortex tissues (n = 5). Mean ± SEM, two-way ANOVA Bonferroni’s post hoc test for multiple comparisons. *p < 0.05, **p < 0.01

In unchallenged *App*^*NL-G-F*^ mice, we observed an increased Aβ deposition at both examined timepoints. Interestingly, 6E10 staining of Aβ revealed that the amount of Aβ plaques is increased in both hippocampus and cortex upon low-grade peripheral inflammation (Fig. 5a, b) and this increase is significant 2 weeks after the second LPS injection compared to PBS injected *App*^*NL-G-F*^ mice. In addition to the amount of Aβ aggregation, also the degree of plaque compactness and the surface area reflect AD pathology [72]. Morphometric analysis of Aβ stained brain sections revealed an increase in small plaques (< 10 µm^2^) in the cortex, while in the hippocampus all different sizes of plaques were increased (< 10 µm^2^, 10–20 µm^2^ and > 20 µm^2^) (Fig. 5c). In agreement with the Aβ plaque analysis, we also observed a significant increase in both soluble and insoluble Aβ_1-40_ in the cortex of *App*^*NL-G-F*^ mice 2 weeks after LPS stimulation (Fig. 5d). For Aβ_1-42_ only the soluble fraction was increased 2 weeks after LPS stimulation (Fig. 5d). However, the levels of Aβ peptides are not significantly changed 24 h after LPS stimulation (Fig. 5d). Taken together, these results suggest that low-grade peripheral inflammation affects Aβ deposition in *App*^*NL-G-F*^ mice, which may be the result of aberrant Aβ clearance from the brain.

### Low-grade peripheral inflammation disturbs Aβ transport across the choroid plexus (CP) epithelial cells

Increased levels of Aβ deposition in the brain may be the result of impaired Aβ clearance that on its term can be the result of a disturbed balance of transport across the brain barriers and/or defective degradation of Aβ aggregates.

**Fig. 6:**
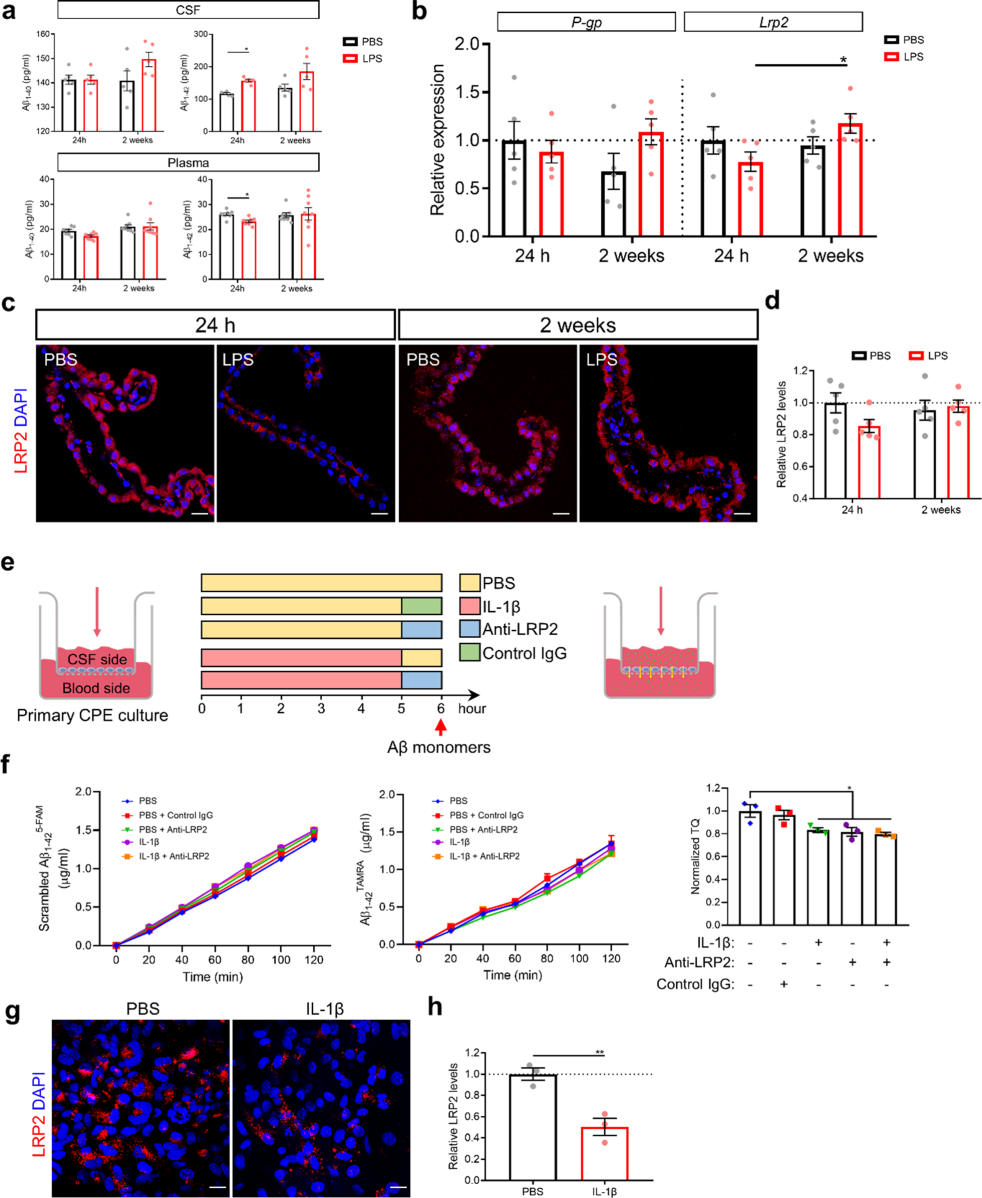
Low-grade peripheral inflammation affects Aβ transport across the blood-CSF barrier in vivo and in vitro. **a** Quantification of Aβ_1-40_ and Aβ_1-42_ in CSF and plasma (n = 5–8). **b** Expression of the genes *P-gp* and *Lrp2* in CP (n = 5). **c** Representative images of LRP2 immunostaining of CP. Scale bar: 20 μm. **d** Quantification of the relative red staining of LRP2 in CP (n = 5 per group). **e** Schematic diagram of Aβ transcytosis analysis in an CP epithelial transport system using fluorescently labeled Aβ peptides. **f** movement of scrambled Aβ_1-42_^5-FAM^ (left) and Aβ_1-42_^TAMRA^ (middle) from the basolateral to the apical chamber, and normalized Aβ transcytosis quotient of Aβ_1-42_ from the basolateral to the apical chamber (right) (n = 3). **g** Representative images of LRP2 staining in primary CP epithelial cells. **h** Quantification of the relative red staining of LRP2 in primary CP epithelial cells (n = 3). Mean ± SEM, two-way ANOVA Bonferroni’s post hoc test for multiple comparisons (**a**, **b**, **d**), one-way ANOVA Bonferroni’s post hoc test for multiple comparisons **f** Nonparametric Mann–Whitney U test **h**. *p < 0.05, **p < 0.01, ***p < 0.001

First, we investigated the effect of low-grade peripheral inflammation on Aβ transcytosis across the blood-CSF barrier. As shown in Fig. 6a, Aβ_1-42_ CSF levels were increased in *App*^*NL-G-F*^ mice 24 h after LPS stimulation. Additionally, the level of Aβ_1-42_ in plasma was significantly lower in *App*^*NL-G-F*^ mice at 24 h, but not at 2 weeks post LPS challenge compared to PBS injected mice (Fig. 6a). To ascertain whether this effect correlates with Aβ efflux from the CSF via the blood-CSF barrier, we analyzed the expression of genes responsible for Aβ influx and efflux in the CP. In line with the changes of Aβ in CSF and plasma, the expression of the Aβ efflux transporters *P-gp* and *Lrp2* showed increased trends 2 weeks post LPS injection compared to the 24 h timepoint in *App*^*NL-G-F*^ mice, and only *Lrp2* showed significant change (Fig. 6b). However, no significant differences were observed compared to PBS injected *App*^*NL-G-F*^ mice. Moreover, protein levels of LRP2 shows a similar trend as the mRNA expression profile (Fig. 6c). Additionally, it has been reported that also LRP1 plays an important role in Aβ transport across CP epithelial cells [15, 47]. Therefore, we looked into the expression of LRP1 in the CP but no obvious changes were seen 24 h after LPS stimulation either at protein nor at mRNA-level in the *App*^*NL-G-F*^ mice (Additional file 4: Fig. 5a-c). Taken together, these data suggest that low-grade peripheral inflammation disturbs Aβ efflux from the CSF into the blood mainly.

Next, we examined whether the observed IL-1β increase in hippocampus during low-grade peripheral inflammation (Fig. 1b) can explain the effect seen on Aβ transport. To address this, we established an in vitro primary CP epithelial transport system using fluorescently labelled Aβ_1-42_ [34, 62]. The addition of fluorescently labelled Aβ_1-42_ monomers to the apical side of the epithelial cells allows the cells to transport the Aβ_1-42_ to the basolateral side. The rate of Aβ transport can be calculated by measuring the transported Aβ_1-42_ in the basolateral compartment. To eliminate the effects of paracellular diffusion of Aβ_1-42_, scrambled Aβ_1-42_ was added to the cell cultures together with Aβ_1-42_ and the transcytosis quotient (TQ) of Aβ_1-42_ was normalized to the diffusion rate of scrambled Aβ_1-42_. Confirming our previous findings, TQ of Aβ_1-42_ was reduced upon IL-1β stimulation of CP epithelial cells (Fig. 6f). IL-1β did not alter the viability of primary CP epithelial cells, even at concentrations ten times greater than that which caused changes in the levels of Aβ transport (Additional file 4: Fig. 6).

To address whether the reduced Aβ transport can be linked to a reduction in LRP2 transport of the Aβ, we first investigated the expression of LRP2 upon IL-1β stimulation. We showed that there is a reduction in LRP2 expression by CP epithelial cells upon stimulation with IL-1β (Fig. 6g, h). The LRP2-dependency of this reduced transport was further studied using a blocking anti-LRP2 antibody. Interestingly, sequential treatment of CP epithelial cells with IL-1β and anti-LRP2 showed no additive effect on Aβ transport (Fig. 0.6f). Importantly, neither the anti-LRP2 antibody nor the IgG control had detrimental effects on blood-CSF barrier integrity (Additional file 4: Fig. 4a, b). Altogether, these data indicate that the pro-inflammatory cytokine IL-1β blocks Aβ transport at least partially by inhibiting LRP2.

### Low-grade peripheral inflammation affects Aβ phagocytosis by microglial cells

**Fig. 7:**
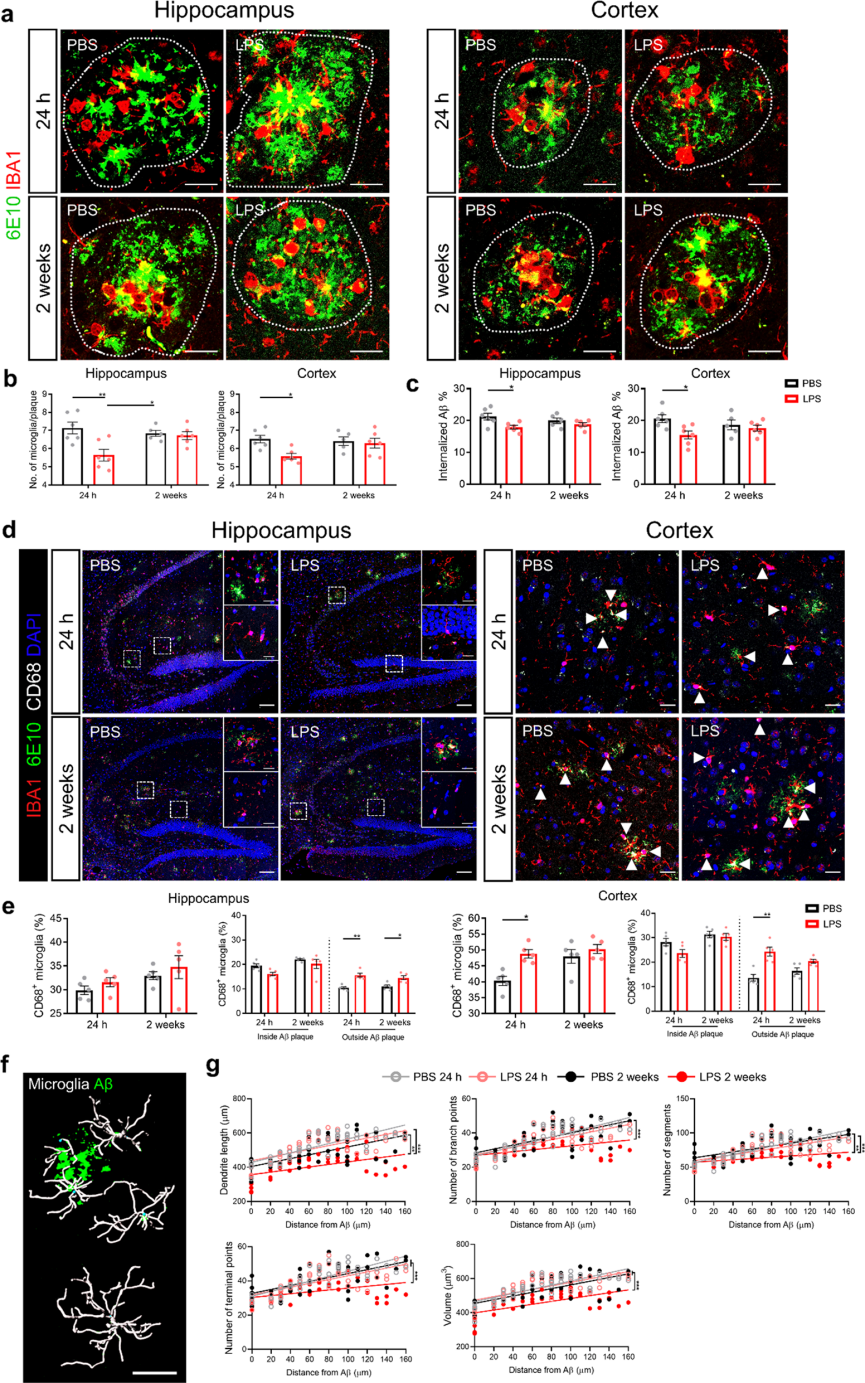
Low-grade peripheral inflammation impairs microglial phagocytosis of Aβ in *App*^*NL-G-F*^ mice. **a** Representative images of IBA1 and 6E10 staining in hippocampus and cortex. The dotted circle shows the border of Aβ plaques. Scale bar: 20 μm. **b** Quantification of the IBA1^+^ microglia within the Aβ plaque surface. Hippocampus (left); cortex (right) (n = 5–6; each symbol represents the average from 4–6 plaques in one mouse). **c** Quantification of the percentage of internalized Aβ in the hippocampus (left graph) and cortex (right) (n = 5–6). **d** Representative images of IBA1, 6E10 and CD68 staining in hippocampus and cortex. Arrow points to the CD68^+^ microglia. Scale bar in hippocampus: 100 μm and 20 μm (insert); Scale bar in cortex: 20 μm. **e** Quantification of CD68^+^ microglia (n = 5) in hippocampus (left two graphs) and cortex (right two graphs). **f** Representative images of relationship between microglial morphology and its distance to Aβ deposition. Scale bars: 20 µm. **g** Imaris-based quantification of cell morphology of IBA1^+^ microglia in hippocampus. Co-staining of IBA1, CD68 and 6E10 was performed on 5 µm paraffin sections. Each symbol represents the average in one cell and 10–15 cells are analyzed per mouse (n = 4–5). Mean ± SEM, two-way ANOVA Bonferroni’s post hoc test for multiple comparisons (**b**,** c**, **e**), nonparametric Mann–Whitney U test **g**. *p < 0.05, **p < 0.01, ***p < 0.001, ****p < 0.0001

Next to Aβ efflux, also defective phagocytotic clearance by microglial cells can explain the increased Aβ deposition in the brain [50]. As shown in Fig. 2, low-grade peripheral inflammation significantly increases microglia proliferation, mainly of the non-plaque-associated microglia. Moreover, the number of microglia around Aβ plaques is reduced 24 h after LPS stimulation in *App*^*NL-G-F*^ mice (Fig. 7a, b). We also found more microglia migration to the vessel at that timepoint (Additional file 4: Fig. 7a, b). Based on these findings, the question arises if the reduced number of microglial cells around Aβ plaques has an effect on Aβ engulfment and clearance. To study this, we quantified the amount of internalized Aβ and observed a decrease in Aβ engulfment by microglial cells 24 h after LPS stimulation in *App*^*NL-G-F*^ mice (Fig. 7a, c). Furthermore, we examined CD68^+^ phagocytic microglial cells by performing a co-staining of CD68, Aβ and IBA1 on hippocampus and cortex (Fig. 7d). A transient increase in CD68 expression is observed 24 h after LPS challenge in the hippocamps and the cortex of *App*^*NL-G-F*^ mice. Interestingly, we observed that less CD68^+^ microglial cells are recruited to the Aβ plaques in *App*^*NL-G-F*^ mice after LPS challenge, especially at the 24-h timepoint (Fig. 7d, e).

Next, we investigated whether the changes in Aβ engulfment can be explained by the activation status of the microglial cells in response to low-grade peripheral inflammation. To study this, we examined the correlation of the activation state of the microglia and the distance to Aβ plaques during low-grade peripheral inflammation. In the hippocampus, our analysis shows that if the microglial cell is in close proximity to Aβ deposits, the LPS stimulation does not lead to further morphological changes of the microglia in terms of dendrite length, cell volume, numbers of segments, branch points and terminal points (Fig. 7f, g). In contrast, peripheral LPS challenge induces a more pronounced microglial activation with increasing distance of the microglia to Aβ plaques compared to PBS injected *App*^*NL-G-F*^ mice (Fig. 7f, g). These morphological changes are reflected by a reduction in dendrite length, cell volume, number of segments, branch points and terminal points. The same morphological changes of microglia in the cortex were observed upon low-grade peripheral inflammation (Additional file 4: Fig. 8).

Altogether, these results show that peripheral inflammation affects microglial activation, migration and motility and reduces microglial Aβ phagocytosis.

### Low-grade peripheral inflammation induces neuronal dysfunction

The phagocytotic ability of a microglial cell is not only important to clear Aβ aggregates, but is also beneficial for the clearance of apoptotic neurons [23, 36]. However, activation of microglia upon peripheral inflammation has shown to cause microglial phagocytosis of healthy neurons and synapses that lead to neuronal loss and dysfunction [41, 58]. With this in mind, we tested whether an enhanced microglial activation and impaired microglial phagocytosis induced by low-grade peripheral inflammation causes neuronal and synaptic loss in *App*^*NL-G-F*^ mice. In line with a previous study [71], we observed increased trends in cell and neuronal death in the hippocampus and cortex after LPS challenge. Yet, neuronal death only reaches statistical significance in WT mice 24 h after LPS challenge (Additional file 4: Fig. 9a-c). Interestingly, the *App*^*NL-G-F*^ mice showed a significant higher percentage of neuronal death in the hippocampus compared to WT mice 2 weeks after PBS injection (Additional file 4: Fig. 9a-c).

**Fig. 8:**
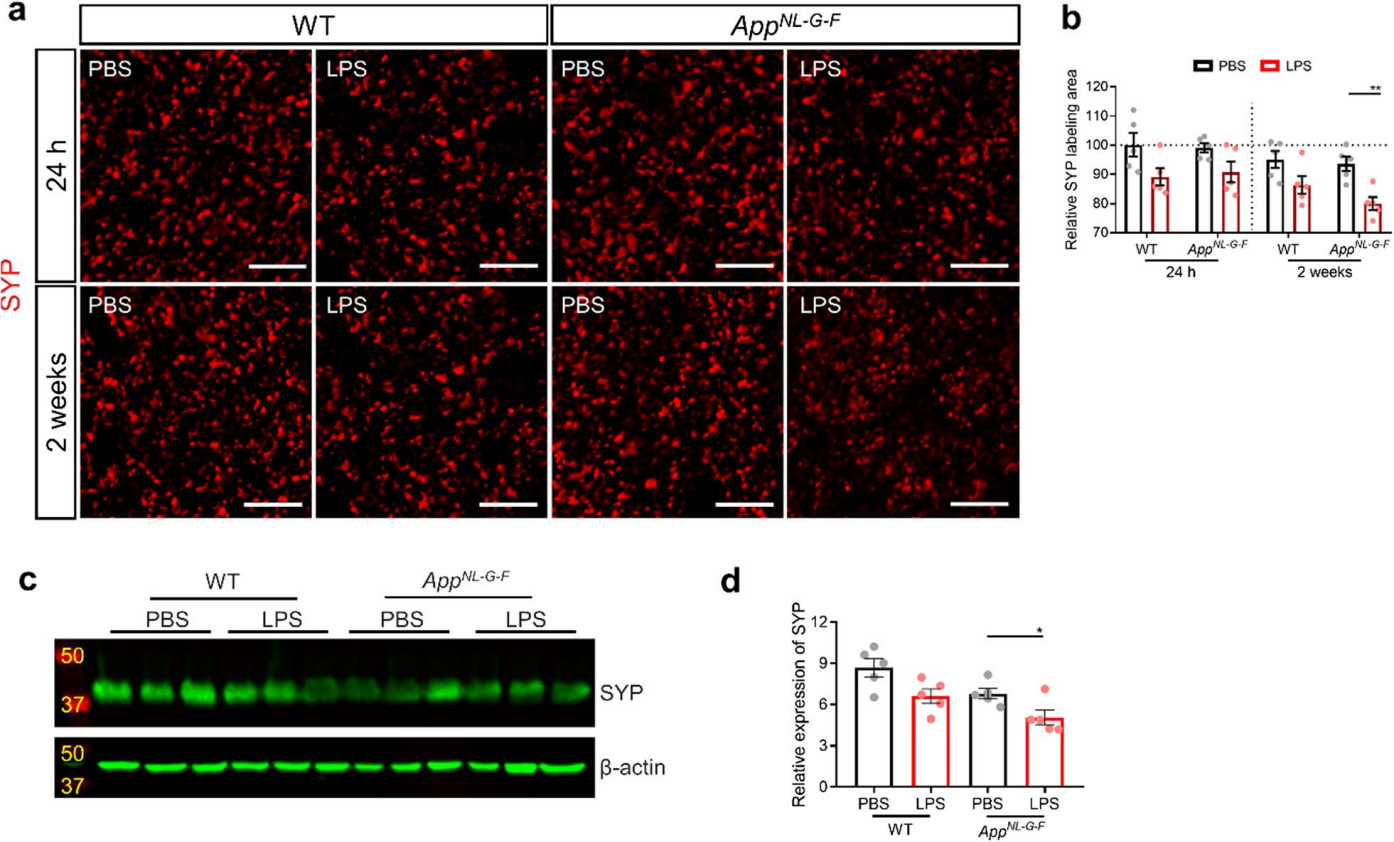
Low-grade peripheral inflammation and Aβ induces neuronal dysfunction. **a** Representative images of SYP staining in the cortex. Scale bar: 10 μm. **b** Quantification of relative SYP^+^ area in cortex (n = 5). **c** Western blot of SYP protein levels in hippocampus 2 weeks after LPS challenge. **d** Densitometric analysis of relative protein levels of SYP normalized to β-actin (n = 5). Scale bar: 50 μm. Mean ± SEM, two-way ANOVA Bonferroni’s post hoc test for multiple comparisons. *p < 0.05, **p < 0.01

In the cortex, immunostainings of the presynaptic marker synaptophysin (SYP) revealed a decrease in expression 2 weeks after low-grade peripheral inflammation in *App*^*NL-G-F*^ mice, but no significant differences at the 24 h timepoint compared to PBS injected *App*^*NL-G-F*^ mice. In WT mice, no LPS-induced obvious differences in SYP expression were observed (Fig. 8a, b). Next, we used western blotting to detect the differences of SYP expression in the hippocampus 2 weeks after LPS stimulation. As displayed in Fig. 8c, d, this confirmed the decrease in SYP expression in the hippocampus upon low-grade peripheral inflammation in *App*^*NL-G-F*^ mice. Correspondingly, coimmunostaining of IBA1 and NeuN showed a higher percentage of microglia and neurons that are in close contact with each other in response to low-grade peripheral inflammation (Additional file 4: Fig. 10a, b).

**Fig. 9:**
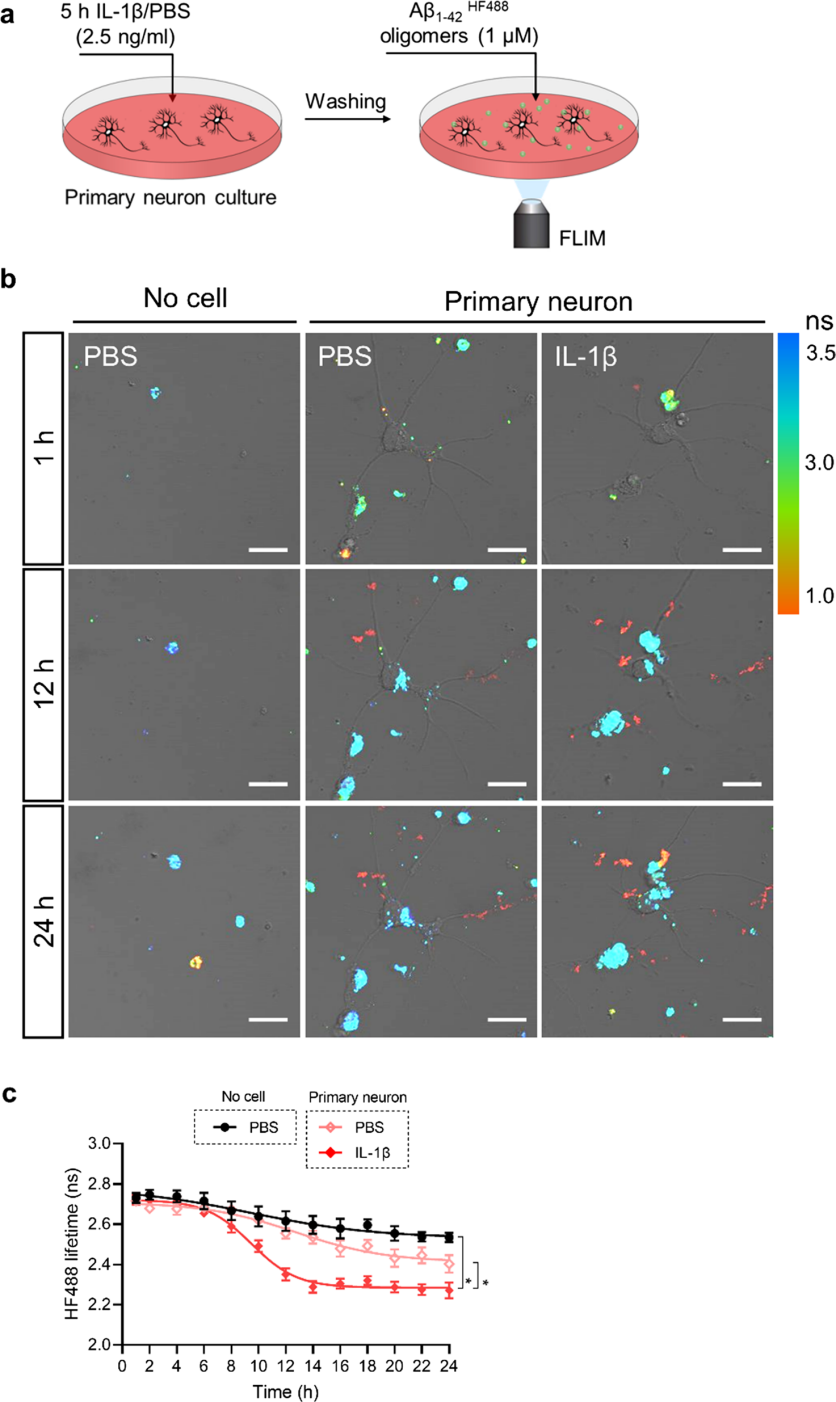
IL-1β stimulation increases Aβ aggregation in primary neuron culture. **a** Schematic diagram of kinetics of Aβ aggregation in primary neuron after stimulation with IL-1β. **b** Representative images of different conditions at 0, 12 and 24 h after adding PBS/Aβ_1-42_^HF488^ oligomers. The images show an overlay of the color-coded FLIM image and the transmitted image. Scale bar: 20 μm. **c** Quantification of HF488 fluorescence lifetime of Aβ_1-42_^HF488^ oligomers (n = 4). Mean ± SEM, nonparametric Mann–Whitney U test. *p < 0.05

Next, we studied whether peripheral inflammation can affect Aβ aggregation by affecting neuron function. Firstly, we performed live cell imaging to monitor the morphologic changes and Aβ biodistribution in primary neuron cultures after IL-1β treatment. Compared to neuronal cell cultures without IL-1β treatment and Aβ (Additional file 1: movie 1), the addition of Aβ affects neuronal function that promotes Aβ aggregation close to the soma (Additional file 2: movie 2). Pretreatment with IL-1β leads to neuronal dysfunction with a reduction in dendrites and a similar Aβ as in the condition where only Aβ was added (without the pretreatment with IL-1β) (Additional file 3: movie 3). However, it can’t be excluded that Aβ aggregation induces fluorescence quenching of HF488-Labeled Aβ_1-42_, reflected by a reduction in fluorescence intensity [13]. To circumvent this phenomenon and study the impact of IL-1β on Aβ aggregation in neurons, we used fluorescence lifetime imaging (FLIM) of HF488-Labeled Aβ_1-42_ to detect Aβ aggregation as described previously [13]. The fluorescence lifetime depends on conformational changes associated with Aβ aggregation and decreases with the increase of Aβ aggregation. As shown in Fig. 9a–c, we observed less and slower Aβ aggregation in the condition without cells compared to Aβ in the presence of neuronal cells. Interestingly, IL-1β treatment further significantly enhances the rate of aggregation in the latter condition. Taken together, these data indicate that neuronal dysfunction affected by peripheral inflammation may play a role in accelerating or exacerbating AD pathology.

## Discussion

Already more than 30 years ago, Aβ was for the first time isolated and proposed to play an important role in AD pathogenesis [18, 19]. Initial efforts in the development of a curative AD treatment mainly focused on strategies to lower Aβ levels and decrease toxic Aβ aggregates. Unfortunately, these attempts are without any clinical success so far, indicating that there is an urgent need for a more in-depth understanding of the onset and progression of AD pathology. Over the last years, it became increasingly clear that the innate immune system plays a crucial role in the pathogenesis of AD, even in the early clinically silent period [17, 24, 64]. Considerable efforts have been devoted to understand the interactions between the peripheral immune system and the CNS [37, 51, 63] in APP overexpression mouse models. However, the underlying mechanisms are not yet fully understood. With this manuscript, we are the first to report the effects of LPS-induced low-grade peripheral inflammation on AD pathology in a second-generation mouse model for AD, namely the *App*^*NL-G-F*^ mouse model. The use of this more representative mouse model may help to give a better understanding of the deleterious impact of peripheral immune activation on AD development.

Tejera et al*.* recently reported that peripheral and CNS inflammation related symptoms return to basal conditions 10 days after a low dose LPS challenge [64]. Based on this study, we used two low-dose LPS injections separated by 7 days to mimic two discrete infectious events associated with low-grade peripheral inflammation. Importantly, IL-6 plasma level analysis revealed a slightly stronger inflammatory response in the* App*^*NL-G-F*^ mice compared to wild type controls and showed in both genotypes a reduced impact of the second compared to the first LPS injection, suggesting partial desensitization. Next, we investigated both the short and long-term effects by analyzing the response 24 h and 2 weeks after the last LPS injection, respectively.

Microglial cells are the most prominent immune cells of the CNS and they continually survey and maintain homeostasis in the brain [23]. Any changes in the CNS lead to activation, proliferation, and morphological changes of these cells [60]. On the one hand, activated microglia can clear neuronal damage by phagocytosis while on the other hand, activated microglia will also release molecules that can initiate neuroinflammation [23]. In concordance with previous findings [64], we observed that microglia located at the site of Aβ deposition don’t show striking morphological changes upon peripheral immune stimulation in contrast to microglia located at a greater distance from the plaques; both in the hippocampus and cortex of *App*^*NL-G-F*^ mice. Indeed, distant microglia showed sustained changes in morphology, reflected by a reduction in dendrite length, cell volume, number of segments, branch points and terminal points. These changes were rather moderate 24 h after the last LPS challenge but further increased after the second LPS challenge. The fact that this is not the case for the microglia in direct contact with the Aβ plaques might be explained by the fact that these microglia are already activated and may not further react to an additional trigger. Next, we showed that the microgliosis marker *Aif1* and microglial activation marker *Cd69* are transiently increased in the hippocampus after LPS challenge. This is however in contrast to a previous study that found that *Aif1* is significantly downregulated in microglial cells upon LPS treatment but is unchanged in the whole brain [63]. Another study reported an increase in *Cd69* expression after exposure of glial cells to LPS or IL-4, but the microglia isolated from CD69^-/-^ mice still exhibited an enhanced production of proinflammatory mediators [6]. Al these results together suggest that an increase in cell number may be responsible for the maintenance or slight increase of *Aif1* mRNA levels and CD69 reported in our study. Moreover, CD69 exerts some negative regulation of inflammation upon exposure to stimulus [6].

The expression levels of the pro-inflammatory cytokines IL-1β and TNF in the brain increase transiently after LPS challenge. These results are consistent with the results of previous studies in first-generation AD mouse models. In those studies, LPS treatment of APP overexpressing mice induces an increased expression of cytokines [37, 64]. However, the extent of the LPS effects are different from our results. For example, a recent study in 5 and 15 months old APP/PS1 mice combined with a single injection of LPS shows high pro-inflammatory cytokine levels 2 days after LPS injection which returns to baseline levels 8 days later [64]. The differences between our results and this study can be explained by a different experimental setup, including LPS type (ultrapure LPS from *S. typhimurium* vs. LPS from *S. abortus equi*, number of LPS injections (single vs. twice, once a week). The different types of LPS may induce varying degrees of inflammatory response and repeated LPS challenge causes less inflammatory response than the first LPS injection. In addition, IL-1β is produced predominantly by microglia and plays an important role in microglia activation and proliferation [36]. Under chronic peripheral inflammation, microglia may reach an effector phenotype that express low levels of cytokines [51]. In our study, we performed two LPS injections to mimic long-term chronic peripheral inflammation and the second stimulus may lead to an exaggerated microglial response. This may explain the results of sustained microglial activation but transient changes in inflammation level in our study.

A pressing question is how pathological insults that reside in circulation and peripheral organs can get into the brain. So far, different mechanisms are proposed among which: transmission by the vagal afferents, peripheral mediators crossing/transporting via circumventricular organs (CVOs) or the BBB, or by signaling through the endothelial cells of the BBB [30]. More recently, the CP epithelial cells that form the blood-CSF barrier are identified as a novel player in this regard [2, 38]. Here, we show that in WT mice, low-grade peripheral inflammation has only limited effects on the blood-CSF barrier integrity, both at short and long-term. This is in contrast to our previous study in which we showed that a single high dose LPS challenge causes severe blood-CSF barrier disturbance [66]. However, long-term peripheral inflammation did induce significant changes in TJ proteins expression and therefore in blood-CSF barrier integrity in *App*^*NL-G-F*^ mice, especially 2 weeks after LPS stimulation. While we previously showed that intracerebroventricular injection of Aβ_1-42_ oligomers contributes to loss of blood-CSF barrier integrity [7, 61], *App*^*NL-G-F*^ mice showed only a trend in increased blood-CSF barrier leakiness compared to WT mice. This might be explained by the low Aβ_1-42_ levels (around 0.15 ng/ml) in the CSF of *App*^*NL-G-F*^ mice which is not sufficient to significantly disrupt blood-CSF barrier integrity compared to the dose of Aβ_1-42_ oligomers (125 ng/ml) injected in our previous studies. In addition, in vitro experiments revealed that IL-1β exposure of primary CP epithelial cells resulted in a significant barrier disruption as previously observed upon TNF stimulation [5]. These results suggest that there are mechanisms in the brain to protect the blood-CSF barrier from peripheral inflammation.

Loss of brain barrier integrity may lead to peripheral immune cell infiltration in the brain via paracellular pathways. In addition, these cells can also cross the brain barriers by the expression of integrin ligands and chemokines by epithelial cells [5]. We identified an increased expression of *Icam1*, *Ccl2* and *Cxcl10*, which are crucial for leukocyte trafficking [5], in the CP and hippocampus of WT and *App*^*NL-G-F*^ mice 24 h after LPS stimulation. These finding are consistent with previous reports [39, 65]. It is therefore important to further understand whether the repeated exposure to a peripheral stimulus ultimately results in the entry of immune cells into the brain. Based on immunohistochemical stainings, we observed an increase in leukocyte infiltration in the brain of both WT and *App*^*NL-G-F*^ mice 2 weeks after the last LPS stimulation. Although we observed an increased infiltration of peripheral immune cells into the brain upon LPS challenges, the infiltration is still very modest. In addition, perivascular macrophages (PVMs) are also IBA1^+^ and TMEM119^−^ and these cells have been shown to increase in neurodegenerative disease models [20]. Therefore, other techniques which can accurately distinguish invading peripheral monocytes from brain endogenous PVMs and microglia, for example using genetic labeling of different myeloid populations [49], should be used to further validate this result. Our results are in agreement with a previous study that showed that a single low dose LPS challenge is sufficient to induce leukocyte infiltration in the brain of aged APP/PS1 mice [64]. Moreover, a sustained exposure triggers a constant influx of leukocytes [65] and this can be caused by brain barrier disruption. On the other hand, one recent study indicates that leukocytes can also pass directly through epithelial cells [42]. Our findings suggest that leukocytes can adapt their behavior to different circumstances as there is no obvious barrier damage in WT mice but still immune cell infiltration.

As microglial activation is associated with Aβ deposition [11], we tested whether in our set-up the low-grade peripheral immune stimulation affects Aβ pathology. This revealed that low-grade peripheral inflammation significantly increases Aβ aggregation and in particular the formation of small size plaques. Previous studies demonstrated that low dose LPS injection(s) lead to increased Aβ deposition in APP/PS1 mice [40, 64] while other studies failed to show this LPS effect in first-generation AD mouse models [31]. The varied outcomes could be due to the experimental setup, including mouse genetic background, age-at-onset of plaque formation, age at LPS stimulation, number of injections, LPS dose, route of administration, and the time between injection and sacrifice. In our study, we found that sustained inflammation stimulates microglia to further transform into a phagocytic phenotype associated with proliferation and morphological changes. This is in line with a previous study which indicated that peripheral inflammation reduces the microglial Aβ clearance [64].

In addition, analysis of microglia biodistribution in the brain suggests that, shortly after induction of peripheral inflammation, microglia migrate to blood vessels, a phenomenon that has been observed before [22]. Consequently, this migration leads to fewer microglia available for Aβ phagocytosis. Two weeks after induction of peripheral inflammation, however, more microglia were again observed close to the Aβ plaques. Nonetheless, Aβ aggregation further continued in response to the chronic low-grade peripheral inflammation, which might be the result of the chronically activated microglial cells which are no longer able to process Aβ [3, 27]. Altogether, our data indicate that peripheral inflammation induces dysfunctional microglia that may account for the increased Aβ deposition which we observed in the brains of LPS challenged *App*^*NL-G-F*^ mice.

Aβ clearance from the brain is also mediated by a combination of transcellular transport mechanisms across the blood–brain and blood-CSF barriers [8, 62]. LRP2 is an efflux transporter expressed at the blood-CSF barrier and is involved in the elimination of Aβ across CP epithelial cells [8]. According to our results low-grade chronic peripheral inflammation induces a short-term effect on Aβ transporter expression levels associated with changes in levels of peripheral and central inflammation. In agreement with this, Ott and colleagues previously suggested that changes in blood-CSF barrier transport are related to the expression of inflammatory cytokines and chemokines, such as higher IL-1β and TNF in serum and CSF of mild cognitive impairment patients are associated with low efficiency of transport small and much larger molecules in the blood-CSF barrier [44]. IL-1β is produced predominantly by microglia and plays an important role in microglia activation and proliferation [36]. To specifically examine the role of IL-1β in the regulation of Aβ efflux, we tested Aβ transcytosis in vitro after IL-1β stimulation. We show that IL-1β not only disrupts the barrier integrity but also affects Aβ transcytosis by inhibiting, in part, the LRP2 transporter. Indeed, we observed that inhibition of LPR2 in CP epithelial cells reduces Aβ efflux from CSF to blood and sequential treatment of anti-LRP2 and IL-1β did not have additive effect on Aβ transport. Altogether, our findings indicate that low-grade peripheral inflammation affects Aβ pathology by multiple pathways including microglial phagocytosis and Aβ transcytosis.

Previous evidence suggests that Aβ plays a vital role in the induction of neuronal dysfunction from synapses toward neural networks [45] and Aβ_1-42_ is considered to be the most neurotoxic form of Aβ [9]. In concordance with this, we found that the soluble Aβ_1-42_ levels were elevated 2 weeks after low-grade peripheral inflammation. Interestingly, we identified synapse loss at the same timepoint and showed that in vitro treatment of primary neurons with Aβ_1-42_ oligomers also induces synapse loss. These results suggest that Aβ_1-42_ may mediate synaptic activity and lead to synapse loss.

Here, we also show that low-grade peripheral inflammation transient induces total cell and neuron death in WT and *App*^*NL-G-F*^ mice. In addition, the *App*^*NL-G-F*^ mice show a higher percentage of neuronal cell death compared to WT mice, which might be due to Aβ pathology induced cell death as demonstrated in previous studies [33, 43]. Microglial cells are important players in the maintenance and plasticity of neuronal circuits, contributing to the protection and remodeling of synapses. The ability of microglial phagocytosis is essential for the clearance of apoptotic neurons and regulation of neuronal activity [23, 36]. In our study, we observed a higher percentage of microglia that are in contact with neurons in short term and long term low-grade peripheral inflammation, which might lead to the removal of pre-synaptic input from neurons as described by Sharma et al*.* [56]. The accumulation of Aβ around synapses has also been shown to induce microglial complement activation in neuronal synapses and subsequent microglial engulfment [4]. Recent study report that TNF induces extracellular Aβ production and aggregation in neuronal cell cultures [70]. Therefore, we hypothesize that pro-inflammatory cytokine IL-1β may be also involved in Aβ aggregation by affecting neuronal functions. In line with this assumption, we found that IL-1β induces accelerated Aβ aggregation in neurons. These findings indicate that peripheral inflammation influences neuronal functioning which subsequently plays an important role in AD pathogenesis.

In conclusion, our study sheds light on how low-grade peripheral inflammation affects AD pathogenesis in a new mouse model of AD, namely the *App*^*NL-G-F*^ model [53], which overcome intrinsic drawbacks of the APP overexpression mouse models by utilizing an *App* knock-in strategy were generated to overproduce pathogenic Aβ such as Aβ_1-42_ without overexpressing APP. In agreement with previous studies in APP overexpression mouse models [31, 40, 57, 64], *App*^*NL-G-F*^ mice showed higher Aβ deposition during low-grade peripheral inflammation. Importantly, we identified several pathways which are activated upon peripheral inflammation and subsequently contribute to aggravation of AD pathology, including sustained microglial activation, neuronal dysfunction and Aβ efflux from the brain. Our results strengthen previous findings in APP overexpressing mice and emphasize the potential vital role of preventing and/or intervening with peripheral inflammation to impact AD development and progression.

## Methods

### Mice

The generation of *App*^*NL-G-F*^ mice carrying Arctic, Swedish, and Beyreuther/Iberian mutations was described previously [52]. The colony of these mice was maintained in our animal house at VIB Center for Inflammation Research. C57BL/6 J mice were used as a control. Mice were kept in individually ventilated cages under a 12-h dark/12-h light cycle in specific pathogen-free animal facility and received food and water ad libitum. Both male and female mice were used and all mice were aged between 20–23 weeks at the start of the experiment. All animal studies were conducted in compliance with governmental and EU guidelines for the care and use of laboratory animals and were approved by the ethical committee of the Faculty of Sciences, Ghent University, Belgium.

### Reagents

LPS from *S. abortus equi* (L-5886) was obtained from Sigma-Aldrich. Recombinant mouse IL-1β was produced in *E*. *coli* and purified by VIB protein core. IL-1β had specific activity of 6.12 × 10^9^ IU/mg and no detectable endotoxin contamination. Synthetic Aβ_1-42_, HiLyte Fluor™ 488-labeled (AS-60479), TAMRA-labeled (AS-60476) and scrambled Aβ_1-42_, 5-FAM labeled (AS-60892) at the N-terminus were purchased from Anaspec. Aβ_1-42_ monomers and oligomers were prepared according to the manufacturer’s instructions.

### Induction of low-grade inflammation

LPS injections (1.0 mg/kg body weight, i.p.) were performed on day 0 and 7 as displayed in Fig. 1a. Control mice received i.p. PBS injections. Depending on the experiment, the mice were sacrificed 24 h or 2 weeks after the second LPS/PBS injection. Body weight, temperature loss, and sickness behavior were checked the first 3 days after LPS injection.

### TLR4 activation analysis

The level of TLR4 activation in plasma and brain was measured using the HEK-Blue mTLR4 assay (InvivoGen) according to the manufacturer’s instructions. HEK-Blue mTLR4 cells were seeded at 25,000 cells per well in a 96-well plate in detection medium. The brain sample was homogenized in PBS supplemented with 1% penicillin/streptomycin (Gibco), and brain supernatant was collected by centrifugation at 20,000*g* in a microcentrifuge at 4 °C for 15 min. The next day, cells were incubated for 12 h with the different samples (20 μl plasma with 1/5 dilutions and 20 μl brain supernatant corresponding to 50 μg total protein), followed by absorption measurement of the culture medium at 655 nm (iMark Microplate Absorbance Reader, Bio-Rad) and relative TLR4 activation was calculated.

### Cytokine measurements

The hippocampus was dissected and immediately snap-frozen in liquid nitrogen and stored at − 80 °C until use. After homogenizing with lysis buffer (0.5% CHAPS in PBS supplemented with complete protease inhibitor (Thermo Scientific)), supernatant was collected by centrifugation at 20,000*g* in a microcentrifuge at 4 °C for 15 min. Protein concentration of the samples was measured using the Pierce BCA Protein Assay Kit (Thermo Scientific). Cytokines in plasma and hippocampus lysates were measured using V-PLEX Custom Mouse Cytokine Kit for IL-1β, TNF and IL-6 (Meso Scale Discovery) according to the manufacturer’s instructions. Signals were measured on a MESO QuickPlex SQ 120 reader (Meso Scale Discovery).

### RNA extraction and RT-qPCR analysis

Samples were snap-frozen in liquid nitrogen and stored at -80 °C until further use. After homogenizing the tissue with TRIzol (Invitrogen) in a tube containing zirconium oxide beads on a TissueLyser (QIAGEN), chloroform was added and the homogenate was separated into 3 phases by centrifugation at 20,000*g* in a microcentrifuge at 4 °C for 15 min. Total RNA was extracted from the upper aqueous phase using Aurum total RNA kit (Bio-Rad) according to the manufacturer’s instructions. The concentration of total RNA was determined by the Nanodrop-1000 (Thermo Scientific) and total RNA was reverse-transcribed into cDNA with SensiFAST™ cDNA Synthesis Kit (Bioline). qPCR was performed on the Roche LightCycler 480 System (Applied Biosystems) using SensiFAST™ SYBR® No-ROX Kit (Bioline). Results are given as relative expression values normalized to the geometric mean of the housekeeping genes. Primers used for qPCR are depicted in Additional file 4: Table 1.

### Measurement of Aβ by ELISA

The Aβ was extracted and measured using a standard protocol as described previously [61]. Briefly, cortex samples were homogenized in Tissue Protein Extraction Buffer containing complete protease inhibitor (Thermo Scientific) and phosphatase inhibitor tablets (Sigma-Aldrich) using a Precellys (Bertin Technologies) and subsequently centrifuged at 5000 g for 5 min at 4 °C. Supernatant was collected and centrifuged at 4 °C for 1 h at 100,000 g (TLA-100 Rotor; Beckman Coulter). Supernatant containing soluble Aβ was removed and stored at − 80 °C. The pellet was further processed in GuHCl solution containing complete protease inhibitor, sonicated, vortexed, incubated for 60 min at 25 °C and centrifuged at 70,000 g for 20 min at 4 °C. Supernatant containing insoluble Aβ was 12 times diluted with GuHCl diluent and immediately frozen at -80 °C. The levels of Aβ_1-42_ and Aβ_1-40_ in plasma, CSF, and cortex lysates were determined using a sandwich ELISA assay. Briefly, the sample-detection mixtures were added to the ELISA plate coated with anti-Aβ_1-42_ (1.5 μg/ml; JRF/cAb42/26) or anti-Aβ_1-40_ antibody (1.5 μg/ml; JRF/cAb40/28) and incubated ON at 4 °C with slow shaking. Absorption at 450 nm was measured after adding substrate solution (BD Biosciences OptEIA™) followed by stopping buffer (1 M H_2_SO_4_). The amount of Aβ was determined with GraphPad Prism 8.0 using a nonlinear regression model.

### Primary mouse CP epithelial cell isolation

Primary mouse CP epithelial cells were isolated from P2-P4 WT pups as previously described [2]. CP tissue was isolated from the lateral and fourth ventricles, pooled and digested with pronase (53,702, Sigma-Aldrich) for 7 min. For monolayer cultures, cells were plated in 24-well Transwell polyester inserts (pore size, 0.4 μm; surface area, 33.6 mm^2^; Corning) coated with laminin (L2020, Sigma-Aldrich). Cells were grown in DMEM/F12 supplemented with 10% FBS, 2 mM L-Glutamine (Gibco), 1% penicillin/streptomycin at 37 °C and 5% CO_2_ for 1 to 2 weeks until their TEER values reached a plateau.

### In vitro*** transcytosis of Aβ***_***1-42***_

The Aβ transcytosis across the epithelial monolayers was measured in triplicate based on the protocol previously described in [21, 34, 62]. In brief, mouse CP epithelial cells (5 × 10^4^ cells per well) were grown to confluence and treated with PBS or IL-1β (2.5 ng/ml) in the basal side of Transwell, followed by 1 h blockage with 15 µg/ml anti-LRP1 or 15 µg/ml control IgG (10500C, Invitrogen). After washing the wells with fresh medium, 1 μM Aβ_1-42_^TAMRA^ monomers and 1 μM scrambled Aβ_1-42_^5−FAM^ monomers in assay medium (DMEM, 5% FBS, 25 mM HEPES (Gibco), 2 mM L-Glutamine) were added to the apical chamber of each well. Subsequently, the cells were incubated at 37 °C. Samples were taken from the basolateral chamber and replaced with fresh medium every 20 min for 2 h and then transferred to 96-well plates (Nunc). The fluorescence was determined using FLUOstar Omega reader (BMG LABTECH) at an excitation wavelength of 485 nm and an emission wavelength of 520 nm. Relative fluorescence units were converted to values of ng/ml, using corresponding standard curves, and were corrected for background fluorescence and serial dilutions over the course of the experiment. Transport of Aβ_1-42_ across the monolayer was calculated asTQ using the following formula:

$$TQ\, = \,{\text{A}}\beta_{{{1} - {42}}} \left( {{\text{d}}Q/{\text{d}}T} \right)/{\text{Scrambled A}}\beta_{{{1} - {42}}} ({\text{d}}Q/{\text{d}}T).$$

where d*Q*/d*T* (μg/s) is the rate of appearance of Aβ on the receiver side after application and is calculated by plotting the cumulative amount (Q) versus time(s).

### Primary neuron culture

Cortical neuronal cells were prepared from embryonic (E16-E18) mouse pups. To dissociate the cortex tissue, the tissue was finely chopped and digested in papain (Sigma-Aldrich) for 20 min. Neurons were triturated gently with a pipette and passed through the 70 µm cell strainer (BD Falcon) before plating them onto Poly-d-Lysine (Thermo Scientific) coated 8-well chamber (iBidi) at a density of 1 × 10^5^ cells per well. Cultures were maintained in Neurobasal medium (Thermo Scientific) containing 1% penicillin/streptomycin, 0.5 mM GlutaMAX™ I Supplement (Thermo Scientific) and 2% B27 supplement (Thermo Scientific) at 37 °C with 5% CO_2_. The absence of astrocytes (< 2%) was confirmed by the virtual absence of glial fibrillary acidic (GFAP) protein immunostaining (data not shown).

#### FLIM

FLIM experiments were carried out using HF488-labeled Aβ_1-42_ oligomers according to a standard protocol as described previously [13]. The images were recorded by time-correlated single photon counting (TCSPC) using Zeiss LSM780 confocal microscope equipped with a MaiTai DeepSee multiphoton laser (SpectraPhysics) and a modular FLIM system (Becker & Hickl). The emitted photons were detected with a hybrid detection module (HPM-100–40). Data was recorded by a Simple-Tau system (M1-SPC-150 FLIM module, Becker & Hickl) with the instrument recording software SPCM version 9.52 in the FIFO image mode using 256 time channels (Becker & Hickl GmbH, Berlin). All acquisitions were done with the 820 nm laser line of the MaiTai DeepSee multiphoton laser with a repetition rate of 80 MHz. A Plan-Apochromat 40x/1.4 Oil DIC objective lens was used and the scanning resolution was set at 512 × 512 pixels. The selected positions were followed for a total of 24 h and every 2 h a FLIM image was captured. All TCSPC images were processed initially using SPCImage version 5.2 (Becker & Hickl) and fitted as mono-exponential decays. Further image analysis was carried out using NIH Image J software. Statistical analysis was performed in GraphPad Software. The average lifetimes in 4 images with healthy cells were computed and these were then averaged to obtain the mean lifetime (± SEM) for each particular time point and treatment. Significant differences between treatments were obtained by means of comparison using nonparametric Mann–Whitney U test.

### Immunohistochemistry

For immunostainings on brain sections, mice were transcardially perfused with ice-cold 4% PFA in PBS. Subsequently, brains were carefully extracted from the skull and split into two halves (mid-sagittal). The right hemispheres were embedded in Frozen Section Medium (Thermo Scientific) immediately in cryomolds (Sakura) that were frozen on dry ice, and stored at − 80 °C until further use. The left hemispheres were post-fixed overnight 4% PFA in PBS at 4 °C. After dehydration, samples were embedded in paraffin in cryomolds and stored at RT until further use. The brains were cut into 5 μm slices paraffin sections (HM 340 E, Thermo Scientific) or 20 μm cryosections (CryoStar NX70, Thermo Scientific). For immunofluorescence staining, sections were permeabilized in PBS containing 0.3–0.5% Triton X-100. Following blocking with goat immunomix (GIM) (5% goat serum, 0.1% BSA, 0.3–0.5% Triton X-100 in PBS) at RT for 1 h, sections were incubated with primary Abs in GIM at 4 °C overnight. After washing with PBS, sections were stained with fluorophore-conjugated secondary Abs in PBS or PBS containing 0.1% Triton X-100 at RT for 1–1.5 h. Counterstaining was done with Hoechst reagent (Sigma-Aldrich, 1:1000 in PBS). For immunostainings on primary cells, cells were fixed with 2% PFA for 20 min on ice. Next, cells were washed three times with PBS and permeabilized with 0.1% Triton X-100 for 10 min on ice. Samples were washed with blocking buffer (1% BSA in PBS) and incubated with primary Abs (diluted in blocking buffer) for 2 h at RT/overnight at 4 °C. After washing with PBS, cells were stained with fluorophore-conjugated secondary Abs in PBS for 1 h at RT. Next, samples were counterstained with Hoechst and the sections were mounted. Confocal laser scanning microscopy was performed using Zeiss LSM780 or Zeiss Axioscan Z.1.

### Image analysis

Quantification was performed using ImageJ software (version 1.53c, National Institutes of Health) and reported as area fraction of region of interest. The expression levels of TJ proteins and transporter proteins were calculated as relative protein positive signal intensity divided by protein positive area. Infiltrating immune cells were calculated as the number of IBA1^+^TMEM119^−^ cells. For inside or outside Aβ plaque-associated microglia quantification, IBA1^+^ cells differentiated by whether they had contact with Aβ plaques and were manually counted. For quantification of the Aβ internalization ratio, the area of Aβ-internalized microglia (Aβ^+^IBA1^+^) was normalized to the Aβ-positive area. The expression of synaptic molecule expression was calculated as percentage of SYP^+^ within the Hoechst area.

### Microglia 3D reconstruction

For 3D reconstruction of microglia, 50 μm vibratome brain sections were stained overnight with anti-IBA1 Ab at 4 °C, followed by secondary Abs for 2 h at RT. Z-stack images were taken with a Zeiss LSM 880 with Fast Airyscan (Zeiss, Germany), using a Plan-Apochromat 40 × 1.3 oil DIC UV-IR M27 objective. The 3D reconstructions and measurements were done by filament tracing algorithm from Imaris software (Bitplane).

### Western blot

Hippocampus extracts were prepared in 0.5% CHAPS buffer containing complete protease inhibitor (Thermo Scientific) and centrifuged for 15 min at 20,000*g* at 4 °C, and protein concentration was determined using a Pierce BCA Protein Assay Kit (Thermo Scientific). Supernatants were denatured in 5xLaemmli buffer, separated by SDS-PAGE gel electrophoresis, and transferred to nitrocellulose. Following blocking with Odyssey Blocking buffer (LI-COR Biosciences), the membrane was first incubated with primary Abs, and then with fluorophore-conjugated secondary Abs. Protein bands were visualized Odyssey Fc Imaging System (LI-COR Biosciences) and quantification was done in Image Studio (LI-COR Biosciences).

### Statistics

Data are shown as mean ± SEM. 2-tailed Student’s *t* test for parametric data was used to compare 2 groups. To compare 3 or more groups, 1-way ANOVA with Bonferroni’s post hoc test for multiple comparisons for parametric data was used. Comparisons of the relationship between the activation state of microglia and the proximity of Aβ plaques between 2 groups were analyzed using nonparametric Mann–Whitney U test. Comparisons of percentages of body weight loss between 2 groups were analyzed using 2-way repeated measures ANOVA. Comparison of 2 factors was analyzed using 2-way ANOVA with Bonferroni’s post hoc test for multiple comparisons for parametric data. GraphPad Prism 8.0 was used for statistical analysis. Differences were considered significant at *P* < 0.05.

## Supplementary Information

**Additional file 1**. Representative movie of primary neuron culture after treatment with PBS for 5 h and traced for 24.h

**Additional file 2**. Representative movie of primary neuron culture after treatment with PBS for 5 h, followed by theaddition of Aβ_1-42_ HF488 oligomers and traced for 24 h.

**Additional file 3**. Representative movie of primary neuron culture after treatment with IL-1β for 5 h, followed by theaddition of Aβ_1-42_ HF488 oligomers and traced for 24 h.

**Additional file 4**. Supplementary Figures S1-S12 and Appendix Tables S1-S2.
