# Supplementary material for: Low-grade peripheral inflammation affects brain pathology in the AppNL-G-Fmouse model of Alzheimer’s disease
Source: Acta Neuropathol Commun. 2021 Oct 7;9:163. doi: 10.1186/s40478-021-01253-z (PMC8499584; doi:10.1186/s40478-021-01253-z)

**Low-grade peripheral inflammation affects brain pathology in the *App^NL-G-F^* mouse model of Alzheimer’s disease**

Junhua Xie^1,2^, Nina Gorlé^1,2^, Charysse Vandendriessche^1,2^, Griet Van Imschoot^1,2^, Elien Van Wonterghem^1,2^, Caroline Van Cauwenberghe^1,2^, Eef Parthoens^3^, Evelien Van Hamme^3^, Saskia Lippens^3^, Lien Van Hoecke^1,2,4^, Roosmarijn E Vandenbroucke^1,2,4*^

^1^ VIB Center for Inflammation Research, VIB, 9052 Ghent, Belgium.

^2^ Department of Biomedical Molecular Biology, Ghent University, 9000 Ghent, Belgium.

^3^ Department of Biomedical Molecular Biology, Ghent University, 9000 Ghent, Belgium; VIB BioImaging Core, VIB, Ghent, Belgium.

^4^ These authors share senior authorship

Address correspondence to Roosmarijn E Vandenbroucke, VIB-UGent Center for Inflammation Research, Technologiepark-Zwijnaarde 71, 9052 Ghent, Belgium.

Phone: + 32 9 33 13730; E-mail: Roosmarijn.Vandenbroucke@irc.VIB-UGent.be

**Conflict of interest statement:** The authors have declared that no conflict of interest exists.

Supplementary Methods

***TEER measurements***

TEER was determined in Z Theta (Applied BioPhysics). Before measurements, both the apical and basolateral chambers were bathed in fresh medium at 37°C. The anode and cathode were carefully immersed in the chambers, and a current was passed across the cell monolayer. Changes in electrical resistance were measured in units of ohm∙cm^2^. The inherent resistance of a blank well coated with 1% fibronectin was subtracted from the obtained values.

***MTT assay***

The untreated and IL-1β treated primary CP epithelial cells were replaced with 100 µl fresh medium and incubated for 4 h with 10 µl of the 12 mM MTT. Added 100 µl of the SDS-HCl solution (1 g SDS in 10 ml of 0.01 M HCl) to each well and mixed thoroughly using the pipette and incubated the microplate at 37°C for 4 h in a humidified chamber. Mixed each sample again using a pipette and read absorbance at 570 nm (iMark Microplate Absorbance Reader, Bio-Rad). Results are presented as percentage of the control values.

***TUNEL and NeuN staining***

TUNEL staining was conducted using a commercial kit (In Situ Cell Death Detection Kit; Roche), following the manufacturer's instructions. In brief, brain sections were incubated with the reaction mixture containing terminal deoxynucleotidyl transferase (TdT) and fluorescein-conjugated deoxyuridine triphosphate (dUTP) for 1 h at 37 °C. After washing with PBS, the sections were blocked and permeabilized in GIM at RT for 1h. Subsequently, these sections were incubated at 4 °C overnight after the addition of primary anti-NeuN antibody and then incubated at RT for 1 h after the addition of secondary antibody.

***Live cell imaging***

The primary neuronal cells were seeded onto a μ-slide 8 well (iBIDI) and cultivated in neurobasal medium with 2% B27. Time-lapse recordings were performed on a spinning disk confocal microscope (Zeiss). This system includes an observer Z.1 microscope equipped with a yokogawa disk CSU-X1. Cells were imaged in a stage-top cell incubator (37 °C with 5% CO_2_) for 24 h with a time interval of 15 min using a pln Apo 20x/0.8 oil DIC II objective and a Photometrics Prime 95B camera.

Supplementary Data

Supplementary Table 1. List of primer sequences used for RT-qPCR analysis.

| **Gene** | **Forward primer sequence (5’-3’)** | Reverse primer sequence (5’-3’) |
| --- | --- | --- |
| *Il1β* | CACCTCACAAGCAGAGCACAAG | GCATTAGAAACAGTCCAGCCCATAC |
| *Tnf* | ACCCTGGTATGAGCCCATATAC | ACACCCATTCCCTTCACAGAG |
| *Il6* | TAGTCCTTCCTACCCCAATTTCC | TTGGTCCTTAGCCACTCCTTC |
| *Aif1* | ATCAACAAGCAATTCCTCGATGA | CAGCATTCGCTTCAAGGACATA |
| *Cd69* | CCCTTGGGCTGTGTTAATAGTG | AACTTCTCGTACAAGCCTGGG |
| *Icam1* | GACCACGGAGCCAATTTCT | GTCAGGGGTGTCGAGCTTT |
| *Ccl2* | TTAAAAACCTGGATCGGAACCAA | GCATTAGCTTCAGATTTACGGGT |
| *Cxcl10* | GACGGTCCGCTGCAACTG | GCTTCCCTATGGCCCTCATT |
| *Zo1* | AGGACACCAAAGCATGTGAG | GGCATTCCTGCTGGTTACA |
| *Zo3* | ACCCTATGGCCTGGGCTTC | CCCGGGTACAACGTGTCC |
| *Cldn1* | GCATGGTGGGAACGCTCAT | CCACAGTCCCTTCAGGTAGGA |
| *Cldn3* | AAGCCGAATGGACAAAGAA | CTGGCAAGTAGCTGCAGTG |
| *Cldn5* | GCAAGGTGTATGAATCTGTGCT | GTCAAGGTAACAAAGAGTGCCA |
| *Cldn11* | ATGGTAGCCACTTGCCTTCAG | AGTTCGTCCATTTTTCGGCAG |
| *Ocln* | CCAGGCAGCGTGTTCCT | TTCTAAATAACAGTCACCTGAGGGC |
| *Cdh1* | CGGACGAGGAAACTGGTCTC | CTTCCGAAAAGAAGGCTGTCC |
| *P-gp* | AGGCCGCACCATTATTTTGTC | GGCAATTCTGTCCCCAAGGAT |
| *Lrp1* | GCTGGGGTGTACGGAAATGG | GTGCTCGAATTTGTTCTGGACT |
| *Lrp2* | TGCCTAAAGGGTTACCCACG | TTGCTGGATTTTGTCCTGGAG |
| *Rage* | CTTGCTCTATGGGGAGCTGTA | GGAGGATTTGAGCCACGCT |
| *Hprt* | AGTGTTGGATACAGGCCAGAC | CGTGATTCAAATCCCTGAAGT |
| *Rpl* | CCTGCTGCTCTCAAGGTT | TGGTTGTCACTGCCTGGTACTT |
| *Ubc* | CTTTCCAGAGAGCGGAACAG | CAAGAACTGCGACCCAAATC |
| *Gapdh* | TGAAGCAGGCATCTGAGGG | CGAAGGTGGAAGAGTGGGAG |

Supplementary Table 2. Antibodies used in specific application.

| Primary Antibody  (Catalog #, manufacture, dilution) | Secondary antibody  (Catalog #, manufacture, dilution) | Section | Application |
| --- | --- | --- | --- |
| Rabbit anti-IBA1  (019-19741, Wako; 1:500) | Alexa Fluor-633 goat anti-rabbit/ Alexa Fluor-488 goat anti-rabbit (A21070, A11008, Thermo Scientific, 1:400) | Paraffin: 5 μm  Cryo: 20 μm  Vibratome: 50 μm | Detection of microglia/macrophages |
| Mouse anti-β-Amyloid, 1-16 (clone 6E10, 803001, BioLegend; 1:500) | Alexa Fluor-568 goat anti-mouse/Alexa Fluor-488 goat anti-mouse (A11004, A11001, Thermo Scientific, 1:400) |  | Detection of Aβ plaques |
| Rabbit-anti-ZO-1  (617300, Invitrogen; 1:500) | Alexa Fluor-633 goat anti-rabbit (A21070, Thermo Scientific, 1:400) | Cryo: 20 μm | Detection of tight junction proteins |
| Rabbit-anti-Claudin-1  (51-9000, Thermo Scientific; 1:200) |  |  |  |
| Mouse-anti-Claudin-5  (35-2500, Thermo Scientific; 1:100) | Alexa Fluor-633 goat anti-mouse (A21052, Thermo Scientific, 1:400) |  |  |
| Mouse anti-E-cadherin  (51-9000, BD Transduction Laboratories; 1:500) |  |  |  |
| Mouse anti-Occludin  (33-1500, Invitrogen;1:100) |  | Paraffin: 5 μm/Cell cultures |  |
| Rat anti-CD31  (DIA-310, Dianova; 1:100) | Alexa Fluor-568 goat anti-rat/Alexa Fluor-633 goat anti-rat (A11077, A21094, Thermo Scientific, 1:400) |  | Detection of blood vessel |
| Rat anti-CD68 (MCA1957GA, Bio-Rad; 1:400) |  |  | Detection of phagocytic microglia |
| Mouse anti-NeuN  (MAB377, Millipore; 1:500) | Alexa Fluor-633 goat anti-mouse/Alexa Fluor-488 goat anti-mouse (A21052, A11001, Thermo Scientific, 1:400) |  | Detection of Neurons |
| Mouse anti-Ki67  (550609, BD Pharmingen; 1:100) | Alexa Fluor-568 goat anti-mouse/Alexa Fluor-488 goat anti-mouse (A11004, A11001, Thermo Scientific, 1:400) |  | Detection of microglia proliferation |
| Rabbit anti-LRP1  (ab92544, Abcam; 1:200) | Alexa Fluor-633 goat anti-rabbit (A21070, Thermo Scientific, 1:400) |  | Detection and blockage of transporters |
| Rabbit anti-LRP-2  (ab76969, Abcam; IHC: 1:200, Transwell: 1:67) |  |  |  |
| Rabbit anti-Tmem119 (ab209064, Abcam; 1:200) | Alexa Fluor-488 goat anti-rabbit (A11008, Thermo Scientific, 1:400) |  | Detection of specific microglia |
| Rabbit anti-Synaptophysin (ab32127, Abcam; IHC: 1:200, WB: 1:20,000) | IHC: Alexa Fluor-633 goat anti-rabbit (A21070, Thermo Scientific, 1:400)  WB: Goat anti-rabbit Dylight 800 secondary antibody (35571, Thermo Scientific; 1:10,000) |  | Detection of synapse |
| Mouse anti-PSD-95  (MA1-045, Thermo Scientific; 1:400) | Alexa Fluor-488 goat anti-mouse (A11001, Thermo Scientific, 1:400) |  |  |
| Mouse anti-β-actin (MA5-15739, Thermo Scientific; 1/10,000) | Goat anti-mouse Dylight 800 secondary antibody (35521, Thermo Scientific; 1:10,000) | / | WB control |

**
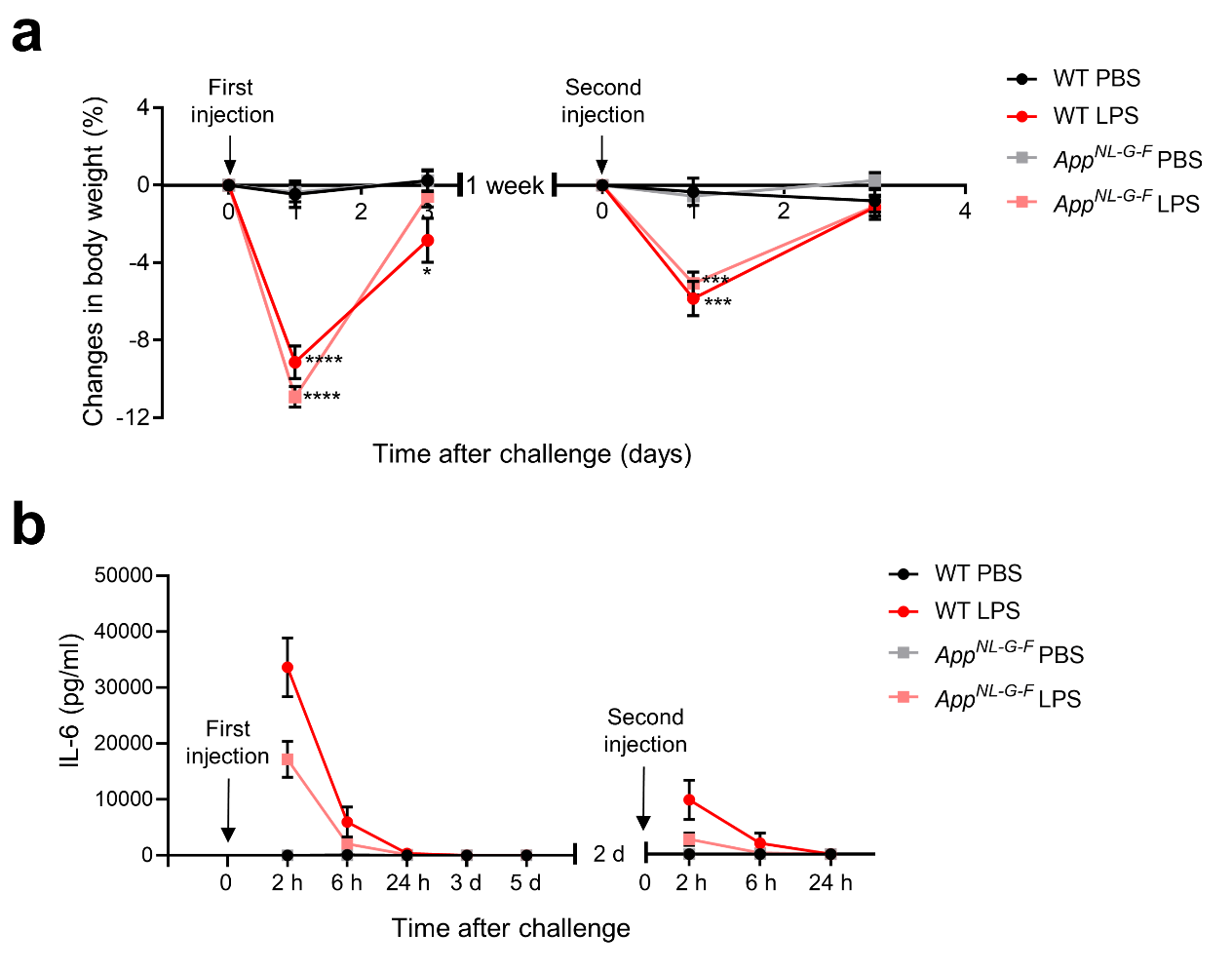
Supplementary Figure 1. LPS-induced peripheral inflammation affects body weight and plasma IL-6 levels. (a)** Change in body weight in WT and App^N-L-GF^ mice at different days upon a first and second low-dose LPS injection. **(b**) IL-6 plasma levels 2, 6, 24 hours, and 3 and 5 days after one and 2, 6 and 24 hours of a second PBS or low dose LPS injection (1 mg/kg) in WT and App^N-L-GF^ mice.


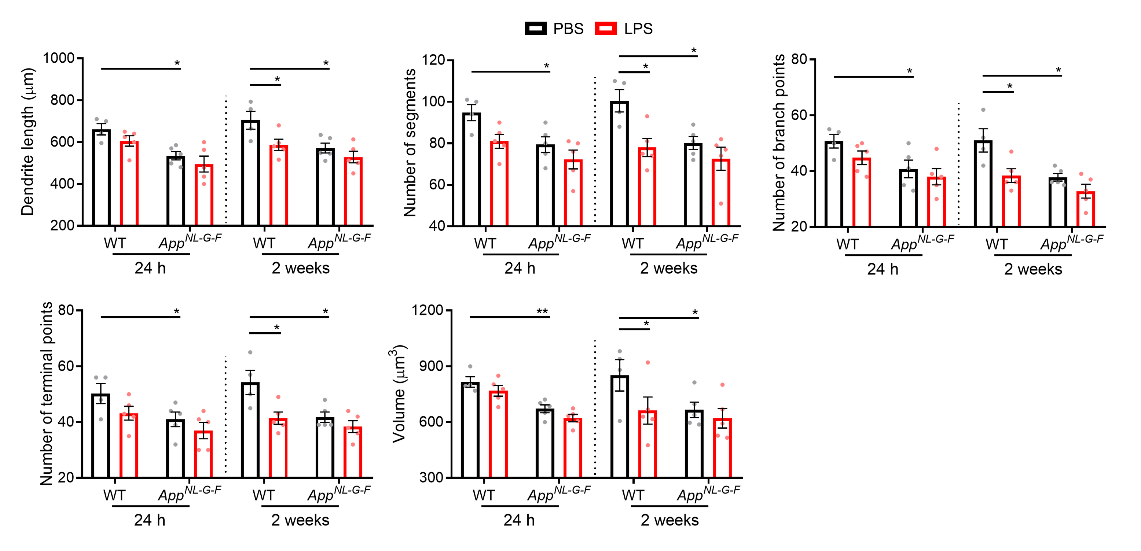


**Supplementary Figure 2. Low-grade peripheral inflammation affects microglia activation.** Imaris-based quantification of cell morphology of IBA1^+^ microglia in cortex. Each symbol represents one mouse, 3-5 cells analyzed per mouse (n=4-5). Mean ± SEM, two-way ANOVA Bonferroni’s *post hoc* test for multiple comparison. **p* < 0.05, ***p* < 0.01.


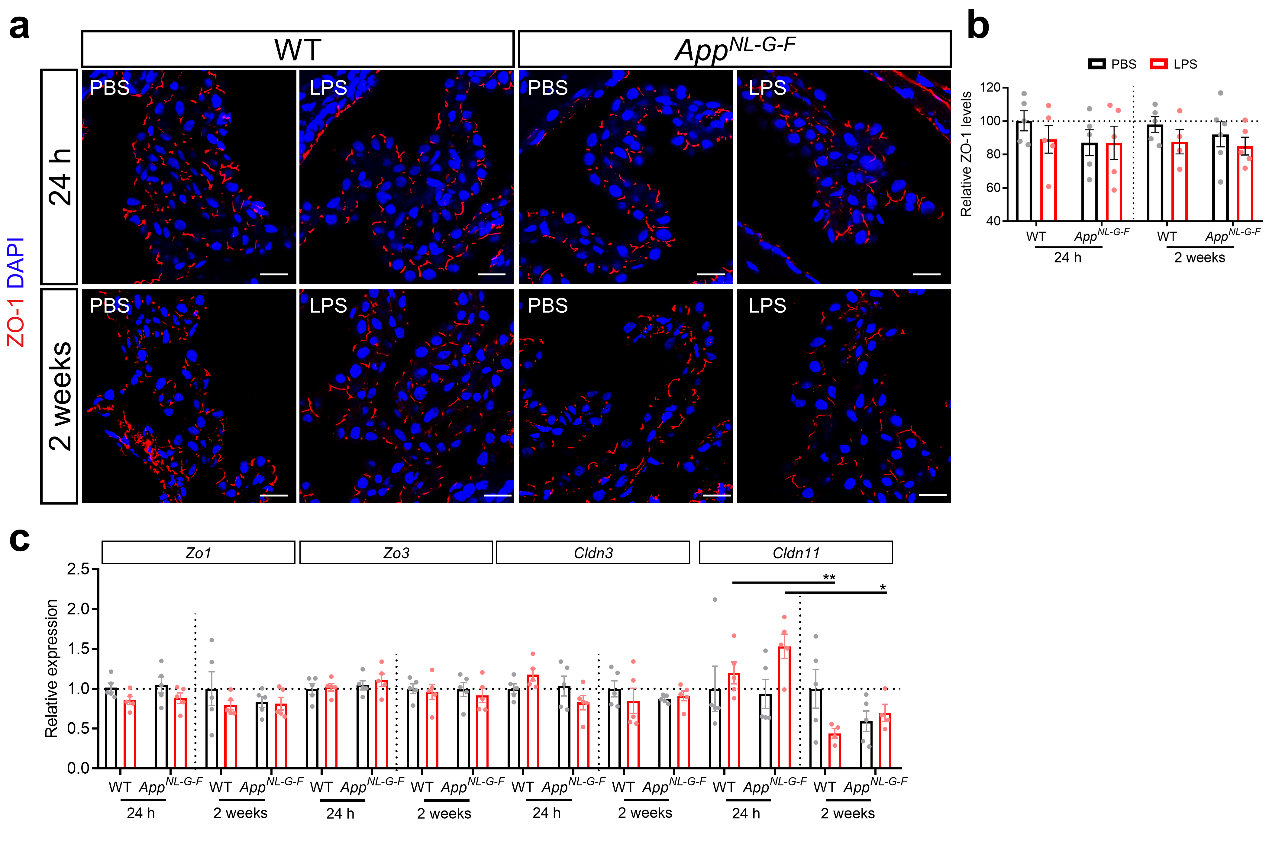


**Supplementary Figure 3. Characterization of blood-CSF barrier integrity during low-grade peripheral inflammation.** (**a**) Representative images of CP stained for ZO-1. (**b**) Quantification of the percentage red staining of ZO-1. (**c**) Relative gene expression of *Zo1*, *Zo3*, *Cldn3,* and *Cldn11* in choroid plexus (n=5 per group). Mean ± SEM, two-way ANOVA Bonferroni’s *post hoc* test for multiple comparisons. **p* < 0.05, ***p* < 0.01.


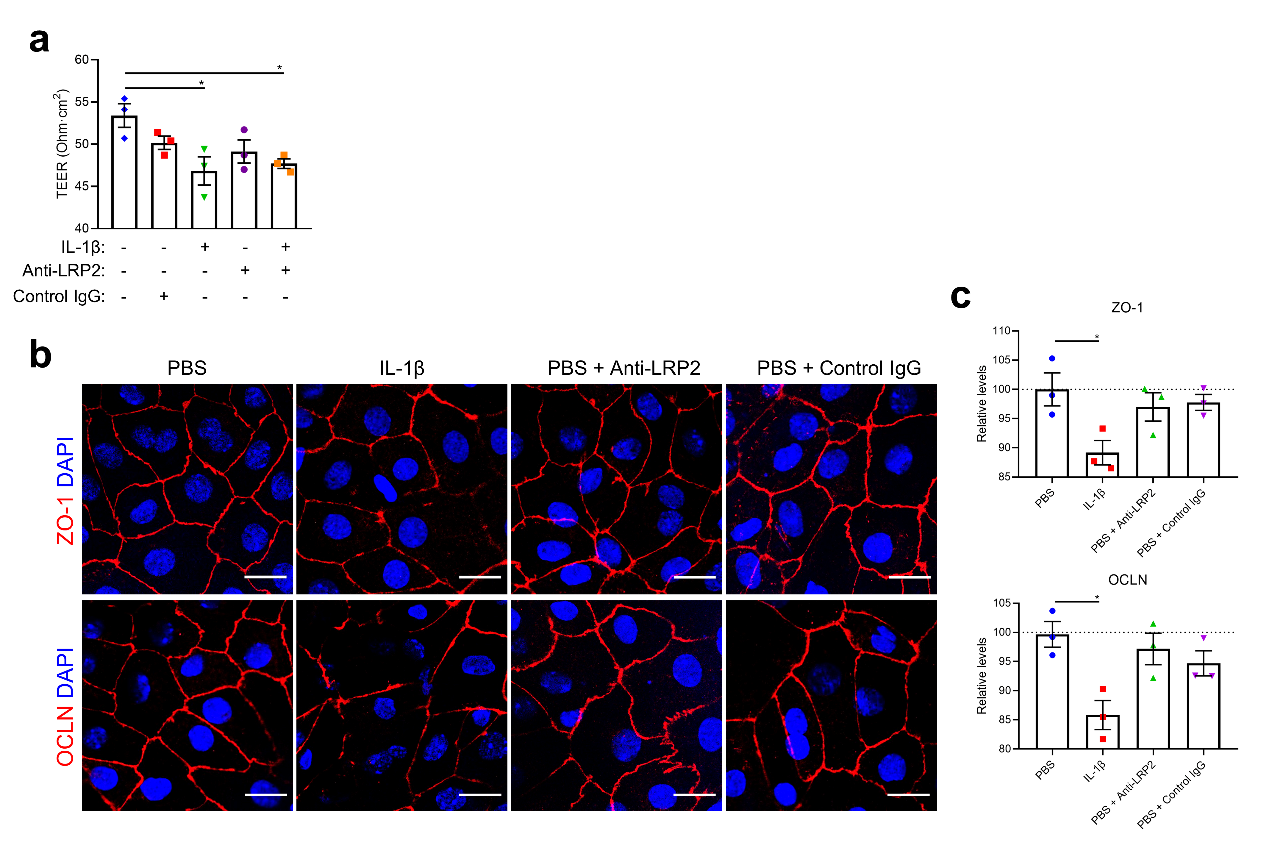


**Supplementary Figure 4. IL-1β disrupts barrier integrity of primary CP epithelial cells.** (**a**) TEER measurements of primary CP epithelial monolayers in Transwell culture after treatment with PBS, IL-1β, PBS + anti-LRP2 or PBS + control IgG (n=3). (**b**) Representative images of ZO-1 and OCLN staining on primary CP epithelial cells after treatment with PBS, IL-1β, PBS + anti-LRP2 or PBS + control IgG. Scale bar: 20 μm. (**c**) Quantification of the percentage red staining of stained for ZO-1 and OCLN (n=3). Mean ± SEM, one-way ANOVA Bonferroni’s post hoc test for multiple comparisons. **p* < 0.05.


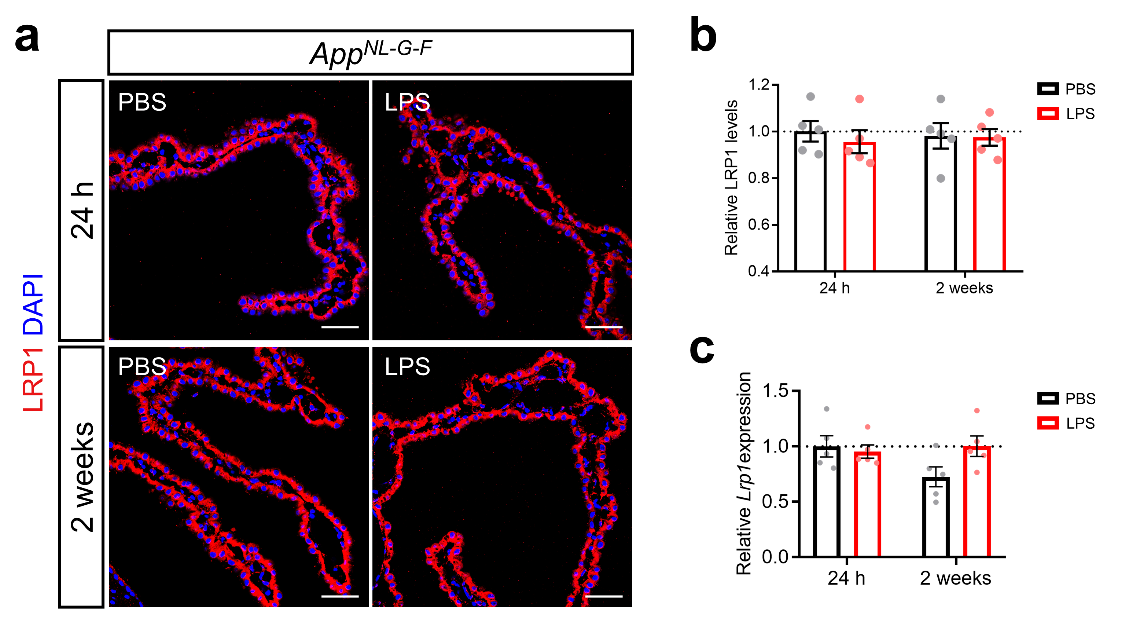


**Supplementary Figure 5. The expression of LRP1 in CP during low-grade peripheral inflammation.** (**a**) Representative images of LRP1 staining in CP. Scale bar: 50 μm. (**b**) Quantification of the percentage of red staining of LRP1 (n=4-5). (**c**) Gene expression of *Lrp1* in CP determined by RT-qPCR (n=5). Mean ± SEM, two-way ANOVA Bonferroni’s post hoc test for multiple comparisons.


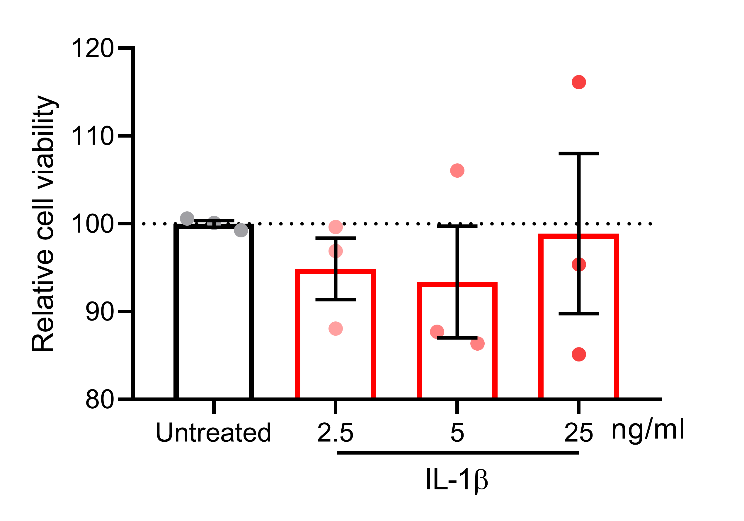


**Supplementary Figure 6.** MTT-based cell viability assay measured 5 h after treatment of primary CP epithelial cells with increasing (2.5 ng/ml-25 ng/ml)concentrations of IL-1β (n=3). Mean ± SEM.


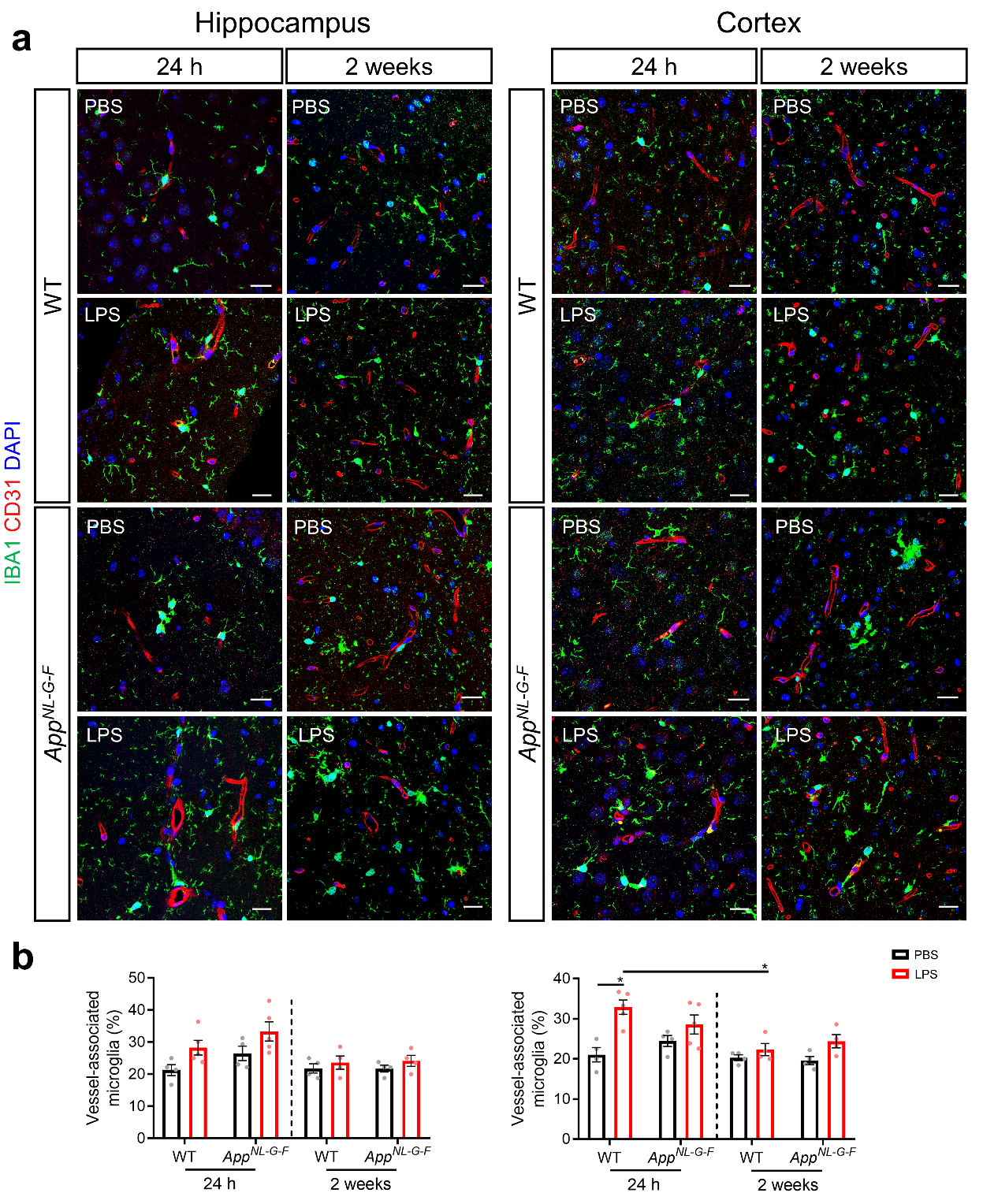


**Supplementary Figure 7. Low-grade peripheral inflammation affects microglia-vessel migration in brain parenchyma.** (**a**) Representative images of CD31 and IBA1 staining in hippocampus and cortex. Scale bar: 20 μm. (**b**) Quantification of the percentage of vessel-associated IBA1^+^ microglia (n=5). Left: hippocampus; right: cortex. Mean ± SEM, two-way ANOVA Bonferroni’s post hoc test for multiple comparisons. **p* < 0.05.


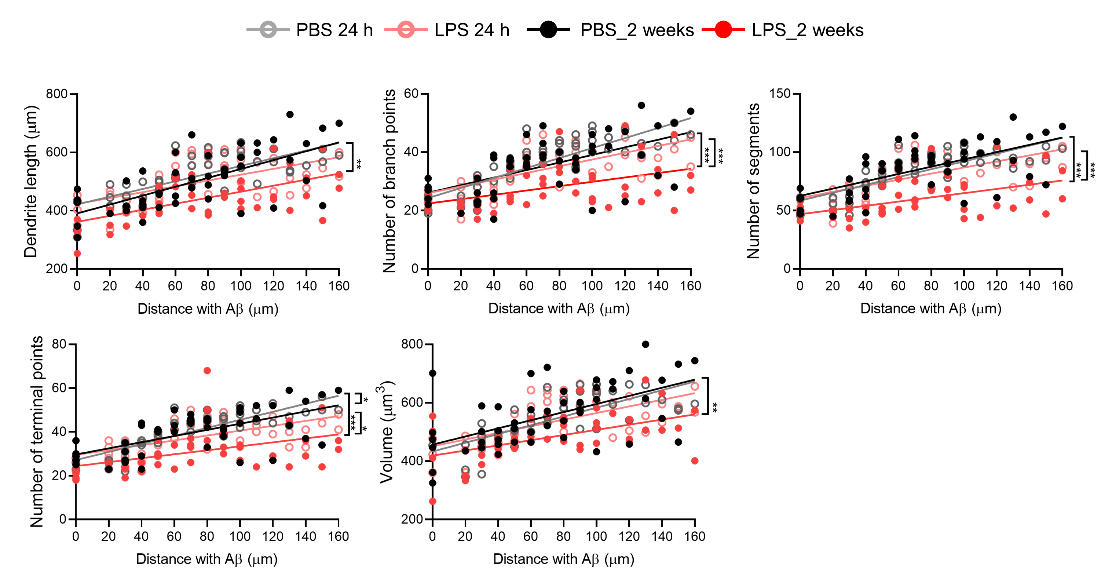


**Supplementary Fig. 8 The effects of low-grade peripheral inflammation and Aβ on microglia activation.** Imaris-based quantification of cell morphology of IBA1^+^ microglia in cortex. Each symbol represents one cell, 10-15 cells analyzed per mouse (n=4-5). Mean ± SEM, nonparametric Mann-Whitney U test. **p* < 0.05, ***p* < 0.01, ****p* < 0.001.


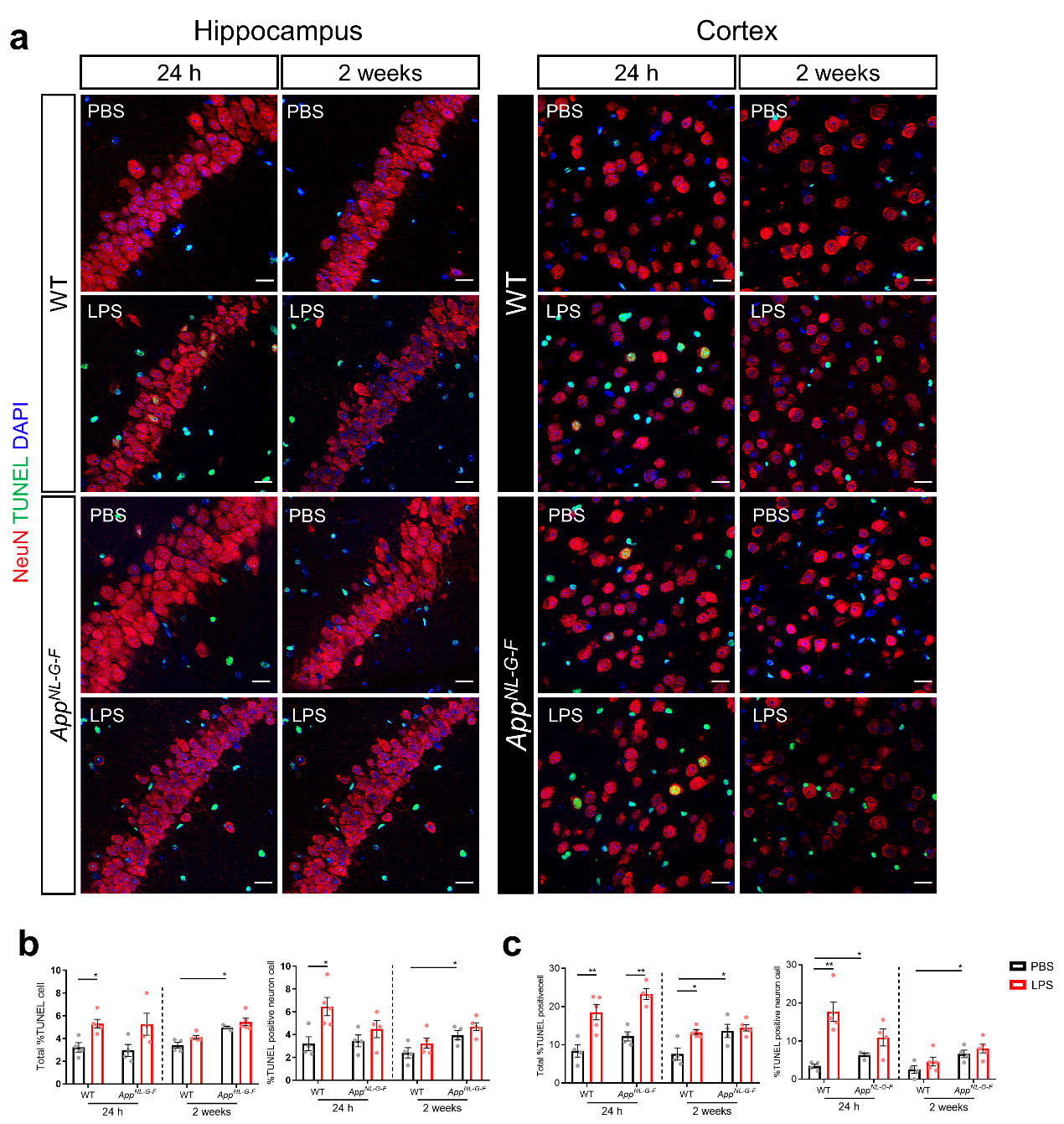


**Supplementary Figure 9.** **Low-grade peripheral inflammation induces cell death.** (**a**) Representative images of TUNEL and NeuN staining in hippocampus and cortex. Scale bar: 20 μm. (**b** and **c**) Quantification of the percentage of total cell death and neuronal cell death in hippocampus (**b**) and cortex (**c**). Mean ± SEM (n=5), two-way ANOVA Bonferroni’s post hoc test for multiple comparisons. **p* < 0.05, ***p* < 0.01.


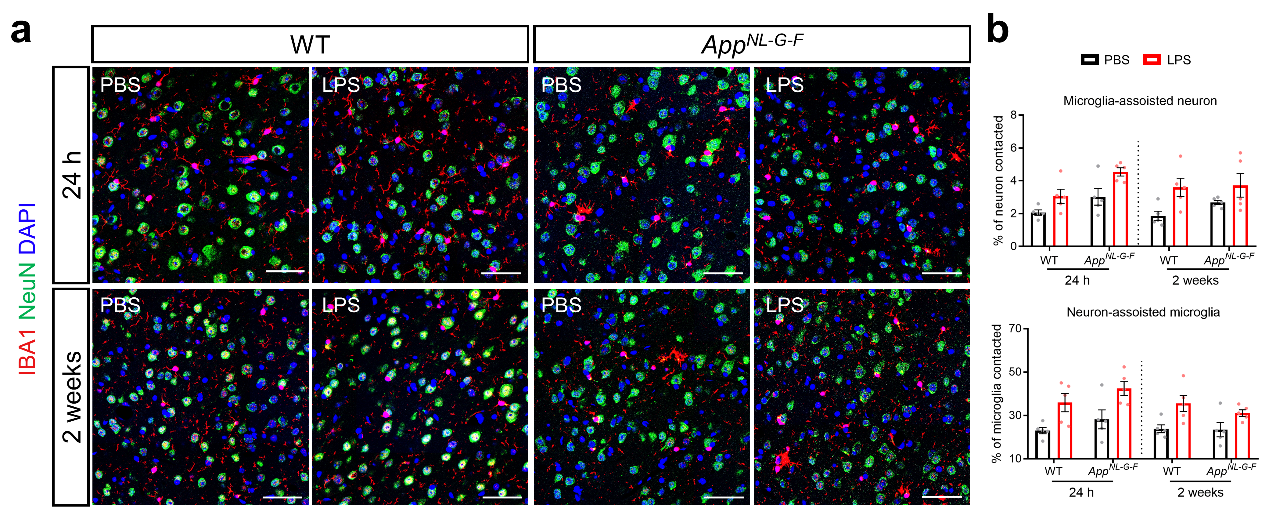


**Supplementary Figure 10. Low-grade peripheral inflammation interaction between microglia and neuron.** (**a**) Representative images of IBA1 and NeuN staining in cortex. Scale bar: 50 μm. (**b**) Quantification of the percentage of microglia-associated neuron and neuron-associated microglia in cortex (n=5). Mean ± SEM, two-way ANOVA Bonferroni’s post hoc test for multiple comparisons. **p* < 0.05.


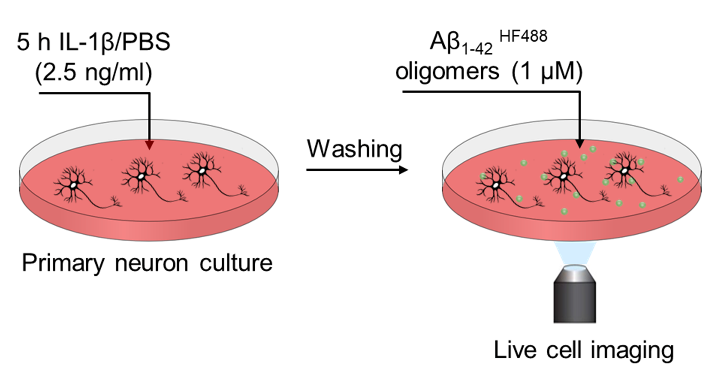


**Supplementary Figure 11.** Schematic diagram of kinetics of neural network activity and Aβ aggregation in primary neuron after immunostimulated with IL-1β.

**Supplementary Fig. 12 Full-length pictures of blots**


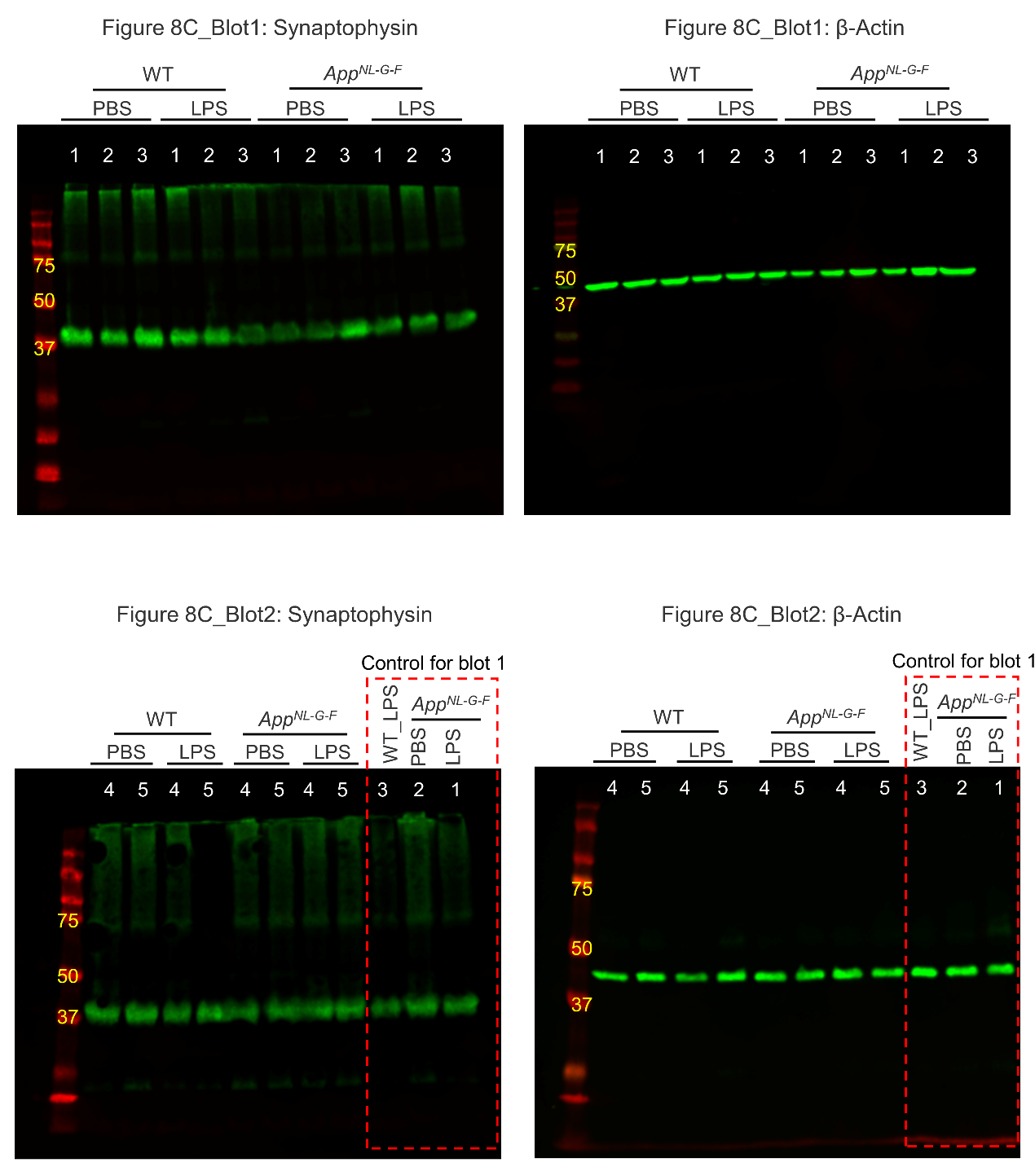

Supplement: Supplementary file 4 — Additional file 4. Supplementary Figures S1-S12 and Appendix Tables S1-S2. [file 40478_2021_1253_MOESM4_ESM.docx]
